# Metastasis-Associated Wound Repair Promotes Reciprocal Lung Epithelium Activation and Breast Cancer Metastatic Outgrowth

**DOI:** 10.1158/2767-9764.CRC-25-0459

**Published:** 2026-04-06

**Authors:** Jessica L. Christenson, Nicole S. Spoelstra, Michelle M. Williams, Linda L. Logan, Kathleen I. O’Neill, David J. Orlicky, Nolan T. Baker, Jennifer A. Wagner, Alyse W. Staley, Adrie Van Bokhoven, Andrew Goodspeed, Li-Wei Kuo, Lyndsey S. Crump, Jennifer R. Diamond, Jennifer K. Richer

**Affiliations:** 1 Department of Pathology, University of Colorado Anschutz Medical Campus, Aurora, Colorado.; 2 Department of Pediatrics, University of Colorado Anschutz Medical Campus, Aurora, Colorado.; 3 University of Colorado Cancer Center Biostatistics Core, Aurora, Colorado.; 4 Department of Biomedical Informatics, University of Colorado Anschutz Medical Campus, Aurora, Colorado.; 5 Division of Medical Oncology, University of Colorado Anschutz Medical Campus, Aurora, Colorado.

## Abstract

When tumor cells colonize distant organs during metastasis, they interact extensively with surrounding cells. These interactions often change the behavior of surrounding cell populations which collectively induce a protumor microenvironment that permits tumor cell outgrowth into overt, clinically detectable metastatic disease. The lung is one of the most common sites of breast cancer metastasis. A chronic wound repair–related phenotype developed within the lung microenvironment during metastatic outgrowth in immunocompetent preclinical mouse models of breast cancer. This phenotype was characterized by an increased number and activation of lung type II alveolar epithelial (AT2) cells surrounding growing metastases. Metastatic outgrowth significantly changed AT2 gene expression, resulting in a modified secretome. AT2-derived secreted factors also promoted triple-negative breast cancer growth. AT2-secreted factors are regulated by the cyclic adenosine monophosphate response element-binding protein (CREB). Targeting CREB signaling with the phosphodiesterase 4 (PDE4) inhibitor roflumilast reduced AT2 breast cancer reciprocal interactions *in vitro* and metastatic outgrowth *in vivo*. Finally, AT2 cells adjacent to metastases in lungs from patients with metastatic breast cancer expressed higher PDE4B compared with AT2 cells in normal lungs.

**Significance:**

Alveolar epithelial cells are the most common cell type in the lung. Our studies demonstrate the potential for targeting metastasis-associated wound repair and lung epithelial cell activation during metastatic outgrowth with FDA-approved PDE4 inhibitors. This strategy may be an effective way to treat and manage progression of established metastatic breast cancer within the lung.

## Introduction

Metastatic outgrowth is one of the most clinically relevant stages of the metastatic cascade (1). During this stage, metastatic disease becomes radiologically detectable, patients are diagnosed, and treatment begins. Although therapeutic interventions used to treat metastatic disease are often based on characterization of a patient’s primary tumor, as many metastases are often difficult to biopsy, there is a significant amount of research indicating that metastases are distinct and may require specifically tailored therapies (2, 3). Thus, it is critical that treatment strategies be developed to specifically target this phase of metastatic progression to reduce the mortality associated with metastases by (i) preventing destructive metastatic outgrowth, (ii) blocking secondary metastatic spread, and (iii) improving metastatic organ function. To achieve this goal, it is imperative that we investigate how the metastatic microenvironment is altered during disease progression and how this supports metastatic outgrowth.

Breast cancer primarily metastasizes to the bone, lung, liver, and brain (4), and the median overall survival for patients with metastatic breast cancer is 1 to 4 years, depending on subtype (5, 6). Approximately one thirds of people with metastatic breast cancer develop lung metastases (7). Aggressive breast cancer, such as triple-negative breast cancer (TNBC), that metastasize within the first few years after diagnosis of primary disease, preferentially metastasize to the lung (8), whereas other subtypes commonly exhibit secondary metastatic spread to the lung from sites like the bone (9). Metastatic breast cancer cells colonize alveoli, the terminal structures of the lung (10) where, as they grow, they cause injury to the alveolus and vulnerable type I alveolar epithelial (AT1) cells. However, the specific contributions of this damage to metastatic progression remain unknown. AT1 cells are responsible for gas exchange and are critical for proper lung function. AT1 cell damage triggers the release of secreted factors that rapidly initiate a wound healing cascade. This cascade is characterized by a distinct series of events, including (i) inflammation caused by the accumulation of neutrophils and macrophages, (ii) the recruitment of fibroblasts that deposit collagen, and (iii) the proliferation of lung type II alveolar epithelial (AT2) cells that terminally differentiate into AT1 cells to repopulate the wound (11).

AT2 cells are often called the “defenders of the alveolus” and have three primary functions, which include: facilitating AT1 gas exchange through the secretion of surfactant that reduces surface tension within the alveolar airspace to prevent lung collapse during breathing, acting as the master regulators of injury and repair within the lung by initiating and coordinating the repair process, and serving as the stem cells of the alveolar epithelium, a necessary component of wound resolution following injury (12). Critical immunologic coordination by AT2 cells is achieved through interactions, both direct or indirect, with innate and adaptive immune cells. AT2 cells have the capacity to recruit neutrophils, activate macrophages, and even present antigen to T cells (as reviewed in ref. 1). AT2 cells are the most numerous cells within the alveolus (13) and are likely to interact extensively with metastatic cells in the lung. Some studies suggest that AT2 cells may play a role in cancer progression (14–17), but none have investigated AT2 behavior in the context of breast cancer metastatic outgrowth. We hypothesized that breast cancer lung micrometastases activate surrounding lung epithelial cells which, in turn, support the outgrowth of metastases.

**Figure 1.**
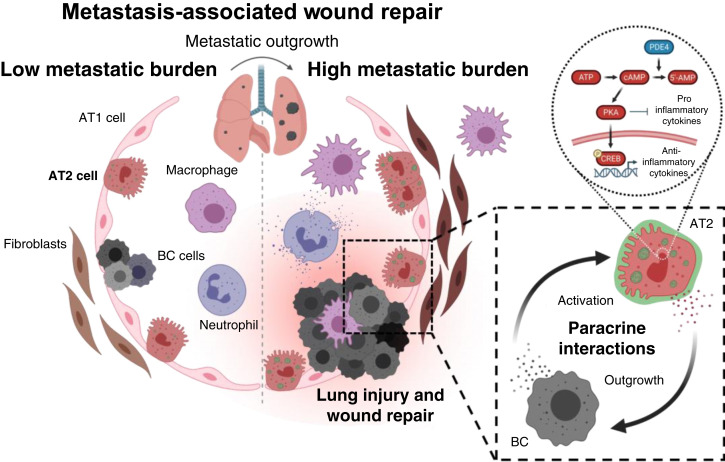
Metastasis-associated wound repair in the lung. Summary model of study results. During metastatic outgrowth, breast cancer (BC) micrometastases grow within the lung alveoli and cause injury to surrounding type I alveolar epithelial (AT1) cells. Chronic wound repair develops throughout this process, which is characterized by an increase in the number and activation of adjacent resident lung cells, including epithelial, immune, and stromal cells. Type II alveolar epithelial (AT2) cells seem to play a prominent role in metastatic outgrowth as a known regulator of wound repair within the lung. During outgrowth, AT2 cells reciprocally interact with adjacent breast cancer cells through paracrine mechanisms. Several AT2-secreted factor genes are known cAMP CREB-regulated genes. PDE4 inhibition may be a rational and effective strategy for blocking cAMP–CREB signaling, AT2-BC interactions, and metastatic outgrowth; ATP, adenosine triphosphate. [Created in BioRender. Christenson, J. (2026) https://BioRender.com/m4okqih.]

Our findings demonstrate that chronic wound repair develops during metastatic outgrowth and that growing metastases alter AT2 cell function in metastasis-adjacent tissue. Activated AT2 cells, in turn, promote metastatic outgrowth through secreted signaling peptides. Most importantly, we were able to impede breast cancer–AT2 reciprocal activation, and consequent metastatic outgrowth, by targeting proinflammatory phosphodiesterase 4 (PDE4) activity using the FDA-approved inhibitor roflumilast (ROF), a drug currently used to treat patients with chronic obstructive pulmonary disease (COPD; Fig. 1).

## Materials and Methods

### Reagents

ROF was purchased from Selleck Chemical LLC. (No. S2131, PubChemCID: 449193), and cilomilast (CILO) from MedChemExpress (No. HY-10790, PubChemCID: 151170). For *in vitro* experiments, ROF and CILO were diluted in dimethyl sulfoxide (DMSO). For *in vivo* experiments, ROF was diluted in a vehicle consisting of 30% polyethylene glycol 400, 0.5% Tween 80, and 5% propylene glycol prepared in sterile deionized water (diH_2_O).

### Cell culture

All cells were cultured in 5% CO_2_. SUM159PT (RRID: CVCL_5423) and MDA-MB-453 (MDA453, RRID: CVCL_0418) TNBC cells were purchased from the University of Colorado Cancer Center (UCCC) Cell Technologies Shared Resource (RRID: SCR_021982) in 2008 and the American Type Culture Collection (ATCC) in 2012, respectively. Both cell lines were maintained in DMEM with 10% fetal bovine serum (FBS). BT549 (RRID: CVCL_1092) TNBC cells were purchased in 2008 from the ATCC and maintained in RPMI 1640 with 10% FBS. MCF7 (RRID: CVCL_0031) estrogen receptor–positive (ER+) breast cancer cells were purchased from the ATCC in 2012 and maintained in MEM with 5% FBS, 1% nonessential amino acids (NEAA), and 6 ng/mL insulin. T47D (RRID: CVCL_0553) ER+ breast cancer cells were kindly provided by Dr. Kathryn Horwitz (University of Colorado) in 2009 and cultured in DMEM with 10% FBS. Aside from A549 (RRID: CVCL_0023) cells, all cells were passaged/subcultured <10 times after thawing. A549 lung carcinoma cells were purchased in 2019 from the UCCC Cell Technologies Shared Resource and maintained in F-12K media with 10% FBS. A549 cells were aged to a more lung AT2 cell–like phenotype by culturing for a minimum of eight passages (18). Human induced pluripotent stem cells (iPSC), originating from male foreskin fibroblasts, were purchased from the Gates Institute at the University of Colorado Anschutz Medical Campus (CU Anschutz) in 2019. iPSCs were maintained on Matrigel-coated plates in mTeSR1 media (Stemcell Technologies, No. 85850) for a maximum of five passages. iPSC cells were differentiated into AT2 cells (iAT2) following the protocol outlined in Tamò and colleagues (19). Briefly, iPSC cells were plated on vitronectin XF (Stemcell Technologies, No. 07180)-coated plates for 3 days in mTerS1 media. iPSCs were differentiated into endoderm cells by culturing in STEMdiff definitive endoderm media (Stemcell Technologies, No. 05110) for 4 days. Endoderm cells were then trypsinized, counted, and plated for experimentation in SAGM media (small airway epithelial cell growth medium; Lonza Walkersville, No. CC-3118) supplemented with 1% FBS for 3 days to induce differentiation into lung AT2 cells. iAT2 cells were cultured for no more than 21 days to avoid transdifferentiation to lung AT1 cells. Met-1 (RRID: CVCL_U373) mouse mammary carcinoma cells were kindly provided in 2015 by Donald McDonnell (Duke University) with permission granted by Alexander Borowsky (University of California – Davis). Met-1 cells were maintained in DMEM with 10% FBS. 66Cl4 (RRID: CVCL_9721) mouse mammary carcinoma cells (20, 21) were kindly provided in 2017 by Heide Ford (University of Colorado) with permission granted by Fred R. Miller (Wayne State University). 66Cl4 cells were maintained in DMEM with 10% fetal calf serum, 1% L-glutamine, and 1% NEAA. All cell lines were routinely tested for *Mycoplasma* contamination and authenticated in 2025 by short tandem repeat DNA profiling by the UCCC Cell Technologies Shared Resource. Transwell plates with 0.4-μm pores were used to investigate paracrine interactions between cell lines (no-contact co-culture), and AT2 media was used for all co-culture experiments. Cell numbers and timeframes for each experiment, depending on cell type and assay, can be found in Supplementary Table S1A.

#### Cell viability

The crystal violet cell viability assay was used to examine how no-contact co-culture or ROF treatment affects cell numbers. Cells were fixed with 10% buffered formalin and stained with 0.1% crystal violet dye prepared in 25% methanol. Stained cells were scanned for analysis of confluence using ImageJ software (NIH, RRID: SCR_003070) to calculate the percent area per well covered by cells. Crystal violet dye was then solubilized with 10% acetic acid, and absorbance was measured at 570 nm to quantify relative cell number. Data are presented as relative cell number normalized to the mean confluence or mean absorbance of cells cultured alone (for co-culture experiments) or DMSO-treated cells (for ROF experiments).

#### Lysotracker stain

For fluorescent imaging, cells were plated in six-well plates, and for fluorescent quantification experiments, cells were plated in 24-well plates. In live cells, nuclei were stained with 2 μg/mL Hoechst for 30 minutes at 37°C. 50 μmol/L LysoTracker Green DND-26 (Cell Signaling Technology, No. 8783), prepared in Opti-Klear Live Cell Imaging Buffer (Abcam, No. ab275939), was then used to stain AT2 lamellar bodies. Representative images were taken using a BX40 microscope, DP73 camera, and cellSens standard software (Olympus, RRID: SCR_014551). Alternatively, fluorescence was measured at an excitation/emission of 485/524 nm for lysotracker and 360/460 nm for Hoechst. Relative to cells cultured alone, co-cultured cell data were presented as the mean lysotracker fluorescence normalized to Hoechst fluorescence.

#### Conditioned media treatments

Conditioned media (CM) were collected from TNBC cells cultured in 10-cm plates for 3 days. Control media were cultured in empty plates for 3 days. Media were collected and cells per plate counted. Media were centrifuged at 1,500 rpm for 4 minutes to remove cellular debris, filter-sterilized, and stored at −80°C. To test the effects of TNBC-secreted factors on AT2 cells, a 1:1 mixture of AT2 media to TNBC CM was added to AT2 cells.

#### Cell morphology

Cells were grown on glass coverslips in six-well plates. Coverslips were sterilized with a wash of 70% ethanol (EtOH), air-dried for 10 minutes, and ultraviolet-exposed for 10 minutes. A549 cells were grown on uncoated coverslips. iAT2 cells were grown on poly-L-lysine (Millipore Sigma, No. P8920)-coated coverslips. At the experimental endpoint, cells were rinsed in 1X phosphate buffered saline (PBS), fixed in 10% formalin for 5 minutes and 50% EtOH for 4 minutes, and washed in 1X PBS for 5 minutes. Cells were hematoxylin and eosin (H&E) stained using an abbreviated protocol. Briefly, coverslips were washed in 100% EtOH for 5 minutes, stained in 1% Eosin-Y (Cancer Diagnostics, Inc., No.832) for 1 minute, washed twice with tap water, stained in Harris Hematoxylin (Cancer Diagnostics, Inc., No.842) for 5 minutes, washed with tap water, and mounted on a slide using Permount mounting medium (Fisher Scientific, No. SP15-100).

### *In vivo* mouse models

*In vivo* experiments were performed in accordance with NIH Guidelines for the Care and Use of Laboratory Animals. All animal experiments were reviewed and approved by the University of Colorado Institutional Animal Care and Use Committee. Mice were euthanized by CO_2_ inhalation followed by cervical dislocation. Tissues were fixed overnight with 10% buffered formalin for histologic analysis. Lungs were intratracheally inflated with formalin prior to dissection to maintain the structural integrity of the lung architecture. Fixed tissues, stored in 70% ethanol, were then processed and paraffin-embedded by the UCCC Pathology Shared Resource (PSR)–Research Histology (RRID: SCR_021994).

#### Transgenic spontaneous metastasis model

Formalin-fixed, paraffin-embedded (FFPE) MMTV-PyMT (mammary-specific polyomavirus middle T antigen) metastatic lung tissue was kindly provided by Susan Kane (City of Hope) in 2014. Tissue was collected from 20-week-old hemizygous, mixed background (C57BL/6, RRID: IMSR_JAX:000664, and FVB) MMTV-PyMT females with multiple mammary tumors.

#### Late-stage metastasis model

To study the latter stages of lung metastasis, such as metastatic outgrowth, 5 × 10^5^ Met-1 mouse mammary carcinoma cells in 100 μL 1X PBS were intravenously injected into the tail veins of 10-week-old female FVB/NJ mice (The Jackson Laboratory, No. 001800, RRID: IMSR_JAX:001800). Alternatively, 3 × 10^5^ 66Cl4 mouse mammary carcinoma cells in 100 μL 1X PBS were intravenously injected into the tail veins of 10-week-old female BALB/cJ mice (The Jackson Laboratory, No. 000651, RRID: IMSR_JAX:001026). To examine how the metastatic microenvironment is altered during outgrowth, lungs with a low versus high metastatic burden were compared. Lungs were collected 1 week following intravenous injection as a model for low metastatic burden and 3 weeks after injection as a model for high metastatic burden. The effects of ROF on metastatic outgrowth were examined using a late-stage metastasis model (*n* = 5–8 mice/group). Mouse weights were measured weekly, and treatment groups were randomized/matched based on weights. Three days after injection of Met-1 cells, mice began daily oral treatment of ROF (5 mg/kg/day) or vehicle control for 3 weeks. The primary endpoint for this experiment was lung metastatic burden.

#### Lung magnetic resonance imaging

To confirm metastatic burden prior to tissue homogenization for single-cell RNA-sequencing (scRNA-seq), the lungs of mice were magnetic resonance imaging (MRI)-scanned by the UCCC Animal Imaging Shared Resource (RRID: SCR_021980). Mice underwent high-resolution breath- and cardiac-gated MRI (22). MRI was performed on anesthetized mice (1.5% isoflurane) using a 9.4 Tesla BioSpec animal MRI scanner (Bruker Medical).

### Histology

Five-μm-thick sections of FFPE tissue samples were used for analyses. Standard procedures were followed for H&E staining using Harris Hematoxylin and Eosin-Y. Neutrophils and macrophages surrounding small versus large metastases were histologically counted by an experienced veterinary pathologist (Linda Kassenbaum Johnson, University of Colorado).

#### Immunohistochemistry

Sections were deparaffinized in a series of xylenes and ethanols. 1X tris-buffered saline (TBS) with 0.05% Tween 20 was used for all washes. Each antibody was optimized to determine the best retrieval buffer, antibody dilution, and detection method. See Supplementary Table S1B for the immunohistochemistry (IHC) protocol details for each antibody used. Antigen heat retrieval was performed using one of three buffers, depending on the antibody: (i) 10 mmol/L citrate buffer, pH 6.0; (ii) 10 mmol/L Tris/1 mmol/L ethylenediaminetetraacetic acid (EDTA), pH 9.0; or (iii) 1X Dako target retrieval solution citrate buffer, pH 6.0 (Agilent Technologies, No. S169984). Antibodies were detected using one of several methods: (i) Vector ImmPRESS anti-rat polymer horseradish peroxidase (HRP; Vector Laboratories, No. MP-7444, RRID: AB_2336530); (ii) Vector ImmPRESS anti-rabbit polymer HRP (Vector Laboratories, No. 7451, RRID: AB_2631198); (iii) Dako REAL EnVision polymer HRP rabbit-mouse (Agilent Technologies, No. K4003, RRID: AB_2630375); and (iv) Jackson biotinylated goat anti-rat IgG (Jackson ImmunoResearch Laboratoies, Inc., No. 112-065-003, RRID: AB_2338168) followed by streptavidin HRP. The ImmPACT DAB peroxidase (HRP) substrate kit (Vector Laboratories, No. ZJ0415) was used for detection. Slides stained by the UCCC PSR–Research Histology for human PDE4 isoforms were processed on a Ventana Benchmark XT Immunostainer and detected using the *ultra*View Universal DAB Detection Kit (Roche Diagnostics, No. 760-500). Cells were counterstained with a 1:5 dilution of Harris Hematoxylin. Slides were mounted using Permount mounting medium. Representative images were taken using a BX40 Microscope (Olympus) with a SPOT Insight Mosaic 4.2 camera and software (Diagnostic Instruments, Inc.).

#### Metastatic burden quantification

To get an accurate representation of metastatic burden following treatment, mouse lungs were serially sectioned, and three sections, separated by 50 μm, were stained for PyMT by IHC. Whole slides were imaged using the Aperio Digital Pathology Slide Scanner (Leica Biosystems) by the UCCC PSR. The number and size (area in μm^2^) of metastases were quantified per slide using the Aperio ImageScope software (Leica Biosystems, RRID: SCR_014311). Data are presented as the average per mouse calculated from all three sections.

#### Picrosirius red staining

Tissues were stained by the UCCC PSR using a standardized protocol. Briefly, slides were deparaffinized in a series of xylenes and ethanols. Slides were stained with Weigert’s Hematoxylin for 8 minutes, washed in tap water for 10 minutes, stained in picrosirius red solution for 1 hour, rinsed with acidified water, and coverslipped with mounting medium. Representative images were taken under white light and polarized light using an Olympus BX51 microscope equipped with a four-megapixel Macrofire digital camera (Optronics) using the PictureFrame Application 2.3 (Optronics).

#### Multispectral immunofluorescence

Sections were deparaffinized in a series of xylenes and ethanols. 1X TBS with 0.05% Tween 20 and diH_2_O were used for washes. See Supplementary Table S1B for protocol and antibody details. Primary antibodies were detected with Opal TSA technology (Akoya Biosciences). Slides were scanned using a Vectra Polaris Quantitative Pathology Imaging System (Akoya Biosciences). Regions of interest were selected for analysis at the periphery of lung metastases to focus on the adjacent microenvironment. Multispectral images were unmixed, and tissue/cellular compartments were segmented before cells were assigned phenotypes using inForm Tissue Analysis software (Akoya Biosciences).

#### Metastatic cell turnover

Kiel 67 (Ki67) and cleaved-caspase 3 (CC3) IHC levels in mouse lung metastases were scored visually in a blinded manner by an expert in histology. The percentage of positively stained cells was determined for each metastasis. Data are presented as the average staining in metastases per mouse.

### Cytokine array

Using the late-stage metastasis model with Met-1 cells, lungs were collected from mice with a low versus high metastatic burden and snap-frozen in liquid nitrogen (*n* = 3 mice/group). The left lobes from each lung were homogenized, and protein was isolated for cytokine analysis using the Proteome Profiler Mouse Cytokine Array Kit (R&D Systems, No. ARY006) according to the manufacturer’s instructions. Protein expression was quantified using ImageJ software and expressed as the mean pixel density per mouse.

### Human lung samples

Deidentified lungs from patients with normal and metastatic breast cancer were provided by the UCCC PSR–Biobanking (RRID: SCR_021989). Protocol approval was obtained from the Colorado Multiple Institutional Review Board, which determined that the study met criteria for a waiver of informed consent under the US Common Rule. Normal lung tissue was collected during autopsy or following traumatic injury. Additional deidentified normal lungs from autopsy were obtained as a tissue microarray from TissueArray.com (No. LCN981). Basic sample demographics can be found in Supplementary Table S1C. PDE4 isoform staining in the metastases, AT2 cells, and lung stromal cells (excluding the AT2 cells) were scored by a board-certified clinical pathologist (L.L. Logan). Only lungs from women ≥20 years of age were included in the comparison analysis between normal and metastatic lungs. IHC scores were calculated by multiplying the percentage of positive cells and the staining intensity, and the combined IHC score for PDE4 was calculated by combining the IHC scores for isoforms A–D.

### Quantitative PCR

QIAshredder columns (Qiagen, No. 79654) were used to prepare cell lysates, and the RNeasy Plus Kit (Qiagen, No. 74136) was used to isolate RNA. cDNA was synthesized using iScript Reverse Transcription Supermix (Bio-Rad, No. 1708840). SYBR Green quantitative gene expression analysis was performed using the PowerUp SYBR Green Master Mix (Thermo Fisher Scientific, No. A45742) on the 7500 Fast Real-Time PCR System (Applied Biosystems). See Supplementary Table S1D for primer details. Relative gene expression was calculated using the comparative cycle threshold method, and values were normalized to *18S*.

### Bioinformatics

#### scRNA-seq

Using the late-stage metastasis model with Met-1 cells, lungs were collected from mice with a low versus high metastatic burden (*n* = 1 mouse/group). Mouse lungs were enzymatically dissociated. Approximately 10,000 cells were used to generate libraries and sequenced by the Genomics Shared Resource (RRID: SCR_021984) at the University of Colorado Anschutz Medical Campus. FASTQ files for each sample were processed using Cell Ranger 2.0.2. (10x Genomics) using a modified version (PYMT was appended) of the mm10 reference genome. The resulting data were then aggregated using the Cell Ranger aggr pipeline. A total of 5,765 cells (high-burden *n* = 3,121; low-burden *n* = 2,644) remained following aggregation. Expression data for high-burden and low-burden cells was imported into an R environment; the data were filtered and analyzed using the R packages Seurat and Monocle. Filtering based on unique gene counts less than 1,000 or greater than 7,000, or mitochondrial percentages greater than 10% resulted in 5,504 cells in the final analysis, with an average of 11,764 unique molecular identifier counts per cell and a median of 2,487 genes per cell. Principal component (PC) analysis was then performed, and the top 10 PCs were selected for subsequent use. The dimensionality was reduced to two dimensions using t-stochastic neighbor embedding, with the 10 PCs being used as input. Cell clusters were demarcated via fast search and find of density peaks (23). Differentially expressed genes (DEG) were identified for each cluster using the two-sided Student *t* test provided by the Seurat FindMarkers function. Normalized gene expression within cells from a particular cluster was compared with expression within cells from all other clusters. Genes with less than a 25% change or those expressed in less than 10% of cells in both populations being compared were excluded from analysis. Following cluster designation, a likelihood ratio test using a generalized linear model was performed to identify genes that vary by cluster. Genes with an adjusted *P* value of 0.05, an average greater than the bottom quintile of averages, and a dispersion higher than what would be expected using the DESeq model (RRID: SCR_014311) were then selected to construct a developmental trajectory using DDRTree. These data are available in the Gene Expression Omnibus (GEO, RRID: SCR_005012) database as GSE319843. Transcriptomics data were then compared with a published tissue dissociation–related gene signature to rule out enzymatic dissociation as a major contributor to gene expression changes between groups (Supplementary Fig. S4A and S4B; ref. 24). Fold changes (FC) are presented as log_2_ differential expression and genes are considered significant with a *P* ≤ 0.05.

#### Bulk RNA-seq

iAT2 cells were no-contact co-cultured with TNBC cells for 5 days. QIAshredder columns (Qiagen, No. 79654) were used to prepare cell lysates, and the RNeasy Plus Kit (Qiagen, No. 74136) was used to isolate RNA. Illumina HiSeq libraries were prepared and sequenced by the Genomics Shared Resource at CU Anschutz, and the UCCC Biostatistics (RRID: SCR_021981) and Bioinformatics (RRID: SCR_021983) Shared Resource assisted with data analysis. Briefly, Illumina adapters and the first 12 base pairs of each read were trimmed using BBDuk (BBMAP; www.sourceforge.net/projects/bbmap), and reads <50 bp after trimming were discarded. Reads were aligned and quantified using STAR (2.6.0a; RRID: SCR_004463) against the Ensembl (RRID: SCR_002344) human transcriptome (hg38.p12 genome release 96). Ensembl IDs were mapped to gene names, and counts of genes with multiple IDs were aggregated. Low-expression genes were removed if mean raw count <1 or mean counts per million (CPM) <1 for the dataset. Reads were normalized to CPM using the edgeR R package (RRID: SCR_012802). Differential expression was calculated using the voom function in the limma R package (RRID: SCR_010943). These data are available in the GEO database as GSE294453. FCs are presented as log_2_ differential expression, and genes are considered significant with an adjusted *P* ≤ 0.05.

#### Gene set overlap

The BioVenn web application (https://www.biovenn.nl/, RRID: SCR_026853; ref. 25) was used to compare gene lists between RNA-seq datasets. Input gene lists included the mouse AT2 DEGs (high metastatic burden vs. low metastatic burden) with *P* ≤ 0.05 and human iAT2 DEGs (co-cultured vs. cultured alone) with adjusted *P* ≤ 0.05. To determine whether AT2 secreted factors are cyclic adenosine monophosphate (cAMP) response element-binding protein (CREB)-regulated, AT2 gene lists were compared with the CREB1 DEG list (CNP0001581) developed by Zheng and colleagues (26).

#### Functional pathway analysis

The NIH DAVID bioinformatics tool (https://davidbioinformatics.nih.gov/, RRID: SCR_001881; refs. 27, 28) was used to perform functional pathway analysis on RNA-seq datasets. Input gene lists included the mouse AT2 DEGs (high metastatic burden vs. low metastatic burden) with *P* ≤ 0.05 and human iAT2 DEGs (co-cultured vs. cultured alone) with adjusted *P* ≤ 0.05. Data include functional annotation clusters with enrichment scores >2 from each gene list that were then compared across all datasets.

#### Upstream regulator analysis

Ingenuity pathway analysis (IPA, RRID: SCR_008653) upstream regulator analysis (29) was used to examine predicted upstream regulators of genes identified by NIH DAVID pathway analysis to be included in the secreted/signaling peptide pathway.

#### Publicly available scRNA-seq analysis

The CZ CELLxGENE Discover online tool (https://cellxgene.cziscience.com; ref. 30) was used to explore single-cell gene expression in publicly available datasets. The GENE Discover collection currently includes 1,338 human and 389 mouse published scRNA-seq datasets. The Tabula Sapiens dataset (GSE201333; ref. 31) was used explore gene expression in human cells, whereas the Tabula Muris dataset (GSE132042; ref. 32) was used to explore gene expression in mouse cells.

### Statistical analysis

An experienced biostatistician (A.W. Staley) from the UCCC Biostatistics Shared Resource assisted with data analysis. Data are presented as the mean ± standard error of the mean. Statistically significant differences, *P* values ≤ 0.05, were calculated using GraphPad Prism 6.0 statistical software (RRID: SCR_002798) and defined as *, *P* ≤ 0.05; **, *P* < 0.01; ***, *P* < 0.001, and ****, *P* < 0.0001. *P* values were listed in each figure for nonsignificant trends. Differences between two groups were determined by unpaired two-tailed *t* tests with a Welch correction to account for any significant differences in variance between groups. Differences between two groups for multiple targets were determined by multiple unpaired two-tailed *t* tests, with Welch corrections when appropriate, and the false discovery rate controlled using the two-stage step-up (Benjamini, Kreieger, and Yekutieli) method (33). Differences between multiple groups were determined by ordinary one-way ANOVA with Tukey multiple comparisons test. Differences between multiple groups for multiple targets were determined by two-way ANOVA with Sidak multiple comparison test. Simple linear regressions were used to analyze correlative comparisons with the proportion of variance from a linear relationship presented as R^2^. Due to limited sample size, nonparametric tests were utilized to analyze all human clinical data, including the Kruskal–Wallis test for multiple comparisons and the Kolmogorov–Smirnov test for grouped data.

## Results

### Chronic wound repair develops during metastatic outgrowth

#### Wound repair–related cells accumulate in the lung microenvironment during metastatic outgrowth

To study how the metastatic microenvironment changes during outgrowth, we examined the lung directly adjacent to metastases of increasing size. We hypothesized that alterations associated with large metastases, that are not present surrounding small metastases, develop during metastatic outgrowth. By our definitions, small metastases in mice had a diameter of less than 150 μm, which equates approximately to a cluster of 10 cells, and large metastases had a diameter of more than 300 μm (Supplementary Fig. S1A and S1B). To put these sizes into perspective, in patients only lung nodules larger than 5 cm in diameter are clinically recommended for routine follow-up, and the majority of these are found to be nonmalignant (34). Proportionately, human pulmonary nodules are approximately 150x larger than what we have defined as “large” metastases in mice; however, the human lung, based on total lung capacity, is about 6,000x larger than the mouse lung (35). Thus, our “large” metastases are particularly big when considering the actual size of mouse lungs compared with humans.

**Figure 2.**
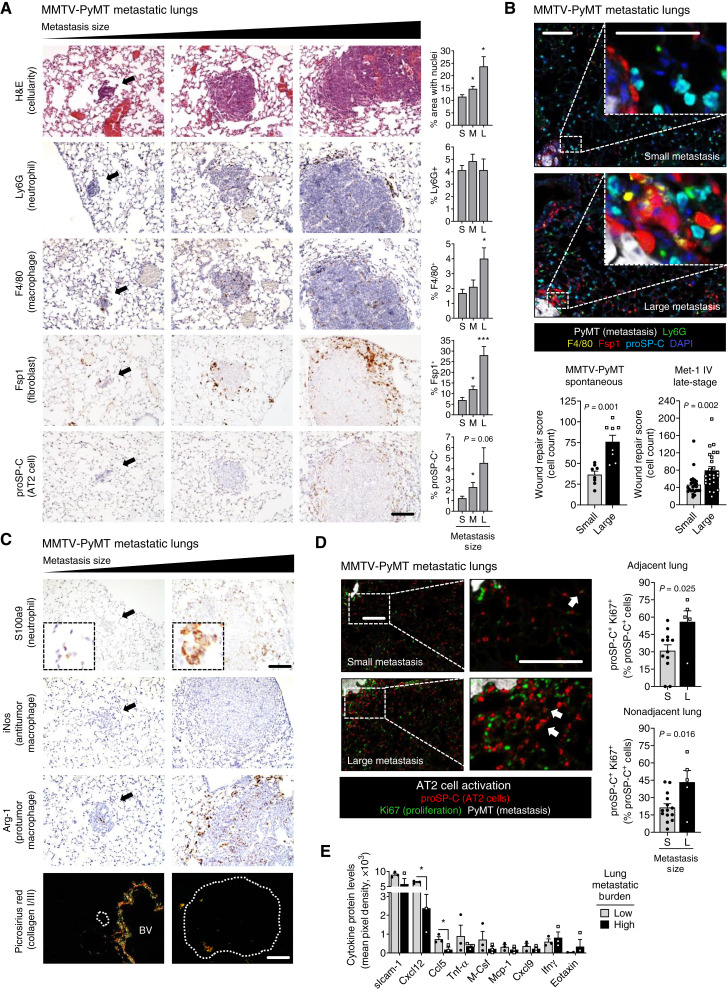
Chronic wound repair in the lung surrounding growing metastases. **A,** MMTV-PyMT metastatic lungs were H&E- and IHC-stained for cell-specific markers of lung wound repair (*n* > 50 metastases from 2–7 mice per stain; see Supplementary Table S1A for sample number details). Shown are representative images from metastases of different sizes, with black arrows indicating small metastases; scale bar, 100 μm. The percentage of positively stained cells, normalized to the total number of cells, was quantified in the 100 μm surrounding metastases. Mean ± SEM (unpaired *t* tests with Welch correction); *, *P* ≤ 0.05; ***, *P* < 0.001. **B,** Multi-IF staining for a lung wound repair cell signature in MMTV-PyMT and Met-1 metastatic lungs. Shown are representative images from MMTV-PyMT metastases of different sizes; scale bars, 25 μm, inset zoom 3×. The wound repair score, calculated as the total number of wound repair–related cells in the 300 μm surrounding metastases from the spontaneous MMTV-PyMT metastasis model (*n* = 16 metastases from 2–4 mice; unpaired *t* tests with Welch correction) and the late-stage Met-1 intravenous (IV) metastasis model (*n* = 50 metastases from five mice; unpaired *t* tests). Mean ± SEM. **C,** MMTV-PyMT metastatic lungs were stained for cell-specific activation markers. Shown are representative images from metastases of different sizes, with black arrows indicating small metastases. For polarized light picrosirius red images, metastases are outlined in white. BV, blood vessel; scale bar, 100 μm, inset zoom 4×. **D,** Multi-IF staining of MMTV-PyMT metastatic lungs for AT2 cell activation. Shown are representative images from metastases of different sizes, with white arrows indicating proSP-C and Ki67 double-positive proliferating AT2 cells; scale bars, 25 μm, inset zoom 3×. The percentage of proSP-C–positive cells that are Ki67-positive was quantified in the lung surrounding metastases: <300 μm surrounding metastases for adjacent cells and >300 μm for nonadjacent cells (*n* = 17 metastases from 3–5 mice; unpaired *t* tests); mean ± SEM. **E,** Cytokine array performed on lungs from mice with a low or high metastatic burden using the late-stage Met-1 IV metastasis model (*n* = 3 mice per group; multiple unpaired *t* tests with Welch correction). Mean ± SEM; *, *P* ≤ 0.05. S, small; M, medium; L, large metastases.

Our initial histologic analysis of the lung metastatic microenvironment in mammary-specific polyomavirus middle T antigen (MMTV-PyMT) transgenic mice showed an increase in the number of cells (cellularization) in the adjacent lung during outgrowth (Fig. 2A; S vs. M *P* = 0.015, S vs. L *P* = 0.020), reminiscent of the epithelization that occurs during wound repair in the lung. We further observed an increase in the number of cells histologically identified as neutrophils and macrophages surrounding small versus large metastases (Supplementary Fig. S1C), cell types that are well-established contributors to wound repair responses. Consequently, to determine whether injury and repair occur in the lung adjacent to metastases during outgrowth, we examined whether cell types classically involved in lung wound repair are present in the lung surrounding metastases using cell type–specific markers. Normal wound repair in the lung is characterized by a cascade of distinct events involving neutrophils, macrophages, fibroblasts, and AT2 cells. Using cell type–specific IHC markers to stain serial sections from MMTV-PyMT metastatic lungs, we observed that the percentage of neutrophils (lymphocyte antigen 6G, Ly6G) surrounding metastases of increasing size remains consistent. The persistent presence of neutrophils throughout outgrowth may be indicative of chronic wound repair stalled during the inflammatory phase (36). In contrast, there was a significant increase in the percentage of macrophages (EGF-like module-containing mucin-like hormone receptor-like 1, F4/80/Emr1; S vs. L *P* = 0.015), fibroblasts (fibroblast-specific protein 1, Fsp1; S vs. M *P* = 0.013, S vs. L *P* = 0.0004), and AT2 cells (prosurfactant protein C, proSP-C; Fig. 2A; S vs. M *P* = 0.038) surrounding large versus small metastases. Notably, these microenvironmental effects were highly localized and only found directly adjacent to metastases within 100 μm of the metastatic perimeter. Very few differences were observed intratumorally (Supplementary Fig. S1D). Our analysis of wound repair–related cells in the lung surrounding metastases indicates that lung compaction, caused by growing metastases, is not solely responsible for what occurs in the lung during metastatic outgrowth, suggesting that cells are actively proliferating and/or being recruited to the metastatic microenvironment (Supplementary Fig. S1E–S1G).

To determine the spatial distribution of wound repair–related cells in the evolving metastatic microenvironment, we developed a multispectral immunofluorescence (multi-IF) wound repair panel with single-cell resolution for staining and cellular localization (Supplementary Fig. S1B). The data from this multi-IF panel reliably recapitulated data obtained from individual IHC stains (Fig. 2A; Supplementary Fig. S2A). By combining the counts for all cell types (neutrophils, macrophages, fibroblasts, and AT2 cells) a single “wound repair score” was calculated to quantify wound repair for each metastasis. Higher wound repair scores were positively correlated with larger metastasis size (Supplementary Fig. S2B). In the MMTV-PyMT spontaneous metastasis model, wound repair is significantly increased in the lung adjacent to large metastases when compared with small metastases (Fig. 2B). Similar results were observed in a late-stage metastasis model in which MMTV-PyMT–derived Met-1 cells were intravenously injected into the tail veins of female FVB/NJ mice (Fig. 2B; Supplementary Fig. S2C). In this model, mammary carcinoma cells directly seed the lung as opposed to spontaneous metastasis models in which mammary carcinoma cells disseminate from the primary tumor. Together, these data indicate that wound repair–related cells consistently accumulate in the lung microenvironment during metastatic outgrowth, irrespective of the presence or absence of a primary tumor or premetastatic niche formation. Interestingly, a comparison of lung tissue surrounding metastases in spontaneous versus late-stage metastasis models indicated that although similar results were observed during metastatic outgrowth, there are significantly more F4/80-positive macrophages present in the lung surrounding small metastases in the late-stage model compared with the spontaneous metastasis model (Supplementary Fig. S2D). This suggests that lung premetastatic niche formation may play a role in spontaneous metastasis by priming alveolar macrophages toward a protumor phenotype. Conversely, alveolar macrophages in the late-stage metastasis model, which have not been primed by a primary tumor, still attempt to clear particulates from the lung, including newly arrived metastatic cells. This multi-IF stain also validated the specificity of our cell type–specific markers. For instance, macrophages can express Fsp1/S100a4 depending on the context (37). In our model there is little overlap between markers, including the macrophages (F4/80), fibroblast (Fsp1), and the mammary tumor PyMT markers (Supplementary Fig. S2E). Finally, our data recapitulated prior studies showing a significant increase in neutrophils and fibroblasts during premetastatic niche preparation when comparing normal lungs to those with a low metastatic burden (38, 39), while also demonstrating that differences are present between the premetastatic (or low metastatic burden) and metastatic (high metastatic burden) niches (Supplementary Fig. S2F and S2G). These data suggest that changes to the microenvironment that arise during metastatic outgrowth are distinct from changes that develop during premetastatic niche formation and/or metastatic colonization.

#### Wound repair–related cells in the metastatic microenvironment become activated throughout metastatic outgrowth

Cell-specific functional markers were used to determine the activation status of wound repair–related cells within the metastatic microenvironment in MMTV-PyMT metastatic lungs. The calcium-binding protein S100a9 is a biomarker indicative of neutrophil activation/stimulation (40). Whereas total neutrophil numbers remained relatively constant during metastatic outgrowth, S100a9 levels were higher in neutrophils surrounding large versus small metastases (Fig. 2C). The increasingly punctate cellular localization of S100a9 in neutrophils throughout metastatic outgrowth is suggestive of increased neutrophil activation. Macrophage polarization and function within the tumor microenvironment are dynamic and extremely complex (41), but the conventional markers used to identify antitumor and protumor macrophage activity are inducible nitric oxide synthase (iNos) and arginase 1 (Arg-1), respectively. Macrophages within the lung metastatic microenvironment were almost exclusively iNos-negative and Arg-1-positive, and the number of Arg-1–positive cells was dramatically increased in the lung surrounding large versus small metastases (Fig. 2C; Supplementary Fig. S3A). The primary function of fibroblasts during wound repair is to secrete collagens. Using the picrosirius red stain for collagens I and III, we found that collagen deposition is low in the lung surrounding metastases, regardless of size (Fig. 2C; Supplementary Fig. S3B). We also examined levels of the traditional fibroblast activation marker α-smooth muscle actin (αSMA). Interestingly, very few αSMA-positive fibroblasts are present in the lung surrounding metastases (Supplementary Fig. S3C). The absence of collagen deposition and αSMA expression are additional indicators of chronic wound repair in the lung (42). Finally, we examined AT2 cell activation, which is traditionally characterized by increased proliferation and transdifferentiation during wound repair resolution (11). Lungs with metastases were multi-IF–stained for the mammary tumor marker PyMT, the AT2-specific marker proSP-C, and the proliferation marker Ki67. AT2 cells surrounding large versus small metastases exhibited significantly more Ki67 staining, indicating increased AT2 proliferation/activation during metastatic outgrowth (Fig. 2D).

Next, we were interested to see whether large metastases influence the microenvironment surrounding other metastases within the same lung. Lungs with large metastases generally have a higher overall metastatic burden (including both large and small metastases; Supplementary Fig. S3D), whereas low metastatic burden lungs exclusively have small metastases. We found that AT2 proliferation was significantly higher in the nonadjacent lung as well as the adjacent lung microenvironments (Fig. 2D). To examine this more closely, we quantified AT2 cell activation in the lungs surrounding small metastases from lungs with a low metastatic burden (S_L_) and small metastases in lungs with a high metastatic burden (S_H_). Interestingly, there were no significant differences in AT2 activation surrounding small metastases regardless of the overall metastatic burden (Supplementary Fig. S3E). These data reflect the complexity of cellular interactions occurring in the lung during metastatic outgrowth. It is possible that distinct effects, or even a gradient of effects, are occurring in cells directly adjacent to metastases compared with cells from more distant lung tissue. Additional in-depth spatial analyses are needed to tease apart these complicated microenvironmental relationships. In either case, our data clearly indicate that AT2 cells are being activated during metastatic outgrowth.

#### A unique cytokine signaling signature is associated with metastatic outgrowth

Cytokines facilitate paracrine communication between cells (43). To identify cytokines associated with metastatic outgrowth and metastasis-associated wound repair, we performed a cytokine array on lungs with low versus high metastatic burden. Whole lung lysates were collected 1 week following intravenously injection as a model for low metastatic burden and 3 weeks after injection as a model for high metastatic burden. Very few differences in cytokine levels were observed when comparing whole lungs with a low versus high metastatic burden, though there was an overall trend in lower cytokine levels with increasing metastatic burden (Supplementary Fig. S3F). Two cytokines, CXC motif chemokine ligand 12 (Cxcl12; low vs. high *P* = 0.022) and CC motif chemokine ligand 5 (Ccl5; low vs. high *P* = 0.039), were significantly reduced in the lung during metastatic outgrowth (Fig. 2E). These cytokines are known to promote breast cancer metastasis and recurrence (44, 45), and both play critical inflammatory roles in wound repair in the lung (46). Interestingly, low levels of CXCL12 and CCL5 are associated with poor wound healing (47, 48). Thus, our results represent an interesting overlap between paracrine signaling related to tumor cell growth and lung wound repair and suggest that cytokine signaling during metastatic outgrowth is unique, particularly when compared with what occurs during tumor cell dissemination and lung seeding. Of note, maximum cytokine diffusion within tissue is estimated to be around 250 μm (49). As the whole lung was examined in this experiment, spatially restricted microenvironmental changes localized directly adjacent to metastases may have been difficult to detect. Nonetheless, these data suggest that chronic wound repair develops in the lung directly adjacent to metastases during outgrowth and that this process may be a unique metastasis-associated response.

### Metastatic outgrowth in the lung induces cell-specific changes in gene expression

**Figure 3.**
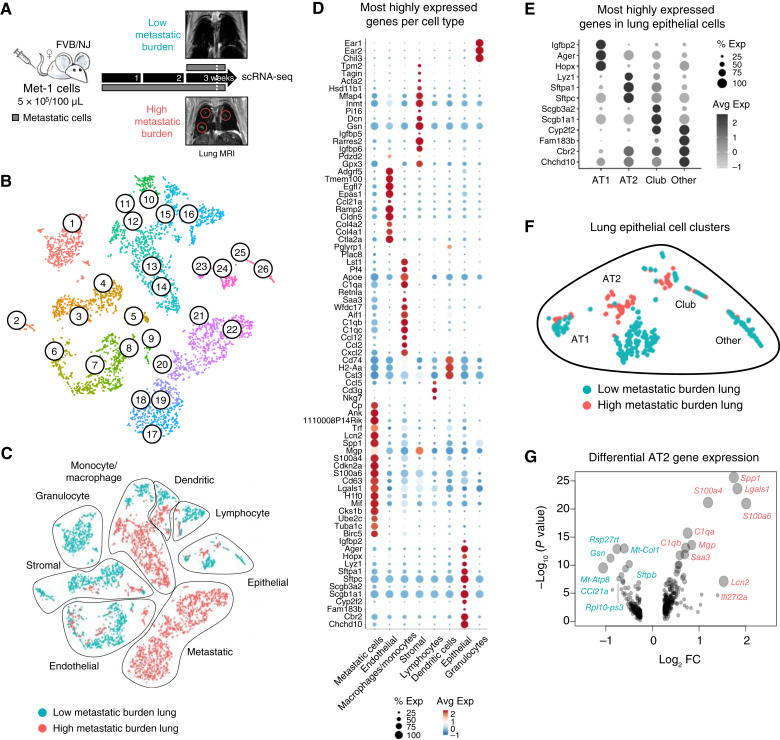
Cell type–specific gene expression within the lung during metastatic outgrowth. **A,** Schematic of scRNA-seq experimental design using the late-stage Met-1 metastasis model. To verify metastatic burden, lungs from mice with a low or high metastatic burden (*n* = 1 mouse per group) were scanned by MRI 3 days prior to tissue collection. Shown are representative coronal MRI images of lungs with low or high metastatic burden, with large metastases circled in pink. Lungs were then collected from perfused mice and enzymatically dissociated for downstream scRNA-seq. **B,** t-distributed stochastic neighbor embedding (t-SNE) visualization of lung cells clustered by gene expression. **C,** t-SNE visualization of cell clusters grouped by cell type and colored by metastatic burden. **D,** Individual cell clusters were grouped by cell type depending on the top 3–5 most highly expressed genes per cluster, using known cell type–specific genetic markers. Average expression (Avg Exp) was defined as average log_2_ FC between one cell type group and all other cell types. Percent expression (% Exp) was defined as the percentage of cells within a cell type group that express each gene. **E,** Epithelial cell clusters grouped by cell type depending on the top 3–5 most highly expressed genes per cluster, using known cell type–specific genetic markers. Club cells, also known as Clara cells, are bronchiolar epithelial cells. **F,** t-SNE visualization of lung epithelial cells clustered by gene expression and colored by metastatic burden. **G,** Differential gene expression in AT2 cells from lungs with a low vs. high metastatic burden. Gene expression displayed by log_2_ FC and −log_10_*P* value. Circle size represents the significance of differential gene expression. Genes labelled in pink were enriched in AT2 cells from lungs with a high metastatic burden, whereas genes labelled in blue were enriched in AT2 cells from lungs with a low metastatic burden.

To examine gene expression changes in the lung microenvironment in response to metastatic outgrowth, we performed scRNA-seq on mouse lungs with low versus high metastatic burden using the Met-1 late-stage metastasis model. MRI with cardiac- and breath-gating was performed to verify lung metastatic burden prior to tissue collection (Fig. 3A). Cellular transcriptomics data were compared with a published dissociation-related gene signature to confirm that the tissue dissociation protocol used was rapid and gentle enough to avoid significant gene expression changes (24). We found that only 65 of 5,504 cells had a dissociation signature greater than the established threshold of 7.5%, suggesting that our enzymatic dissociation technique had little effect on gene expression (Supplementary Fig. S4A and S4B). We first examined organ-wide changes that develop during metastatic outgrowth in the lung by performing a bulk analysis, combining gene expression data from all cell types per condition, and found that peptide signaling, innate immunity, and inflammatory pathways were associated with high metastatic burden (Supplementary Fig. S5A; Supplementary Tables S2 and S3). To examine cell-specific gene expression changes associated with metastatic outgrowth, lung cells were divided into 26 clusters expressing unique gene expression signatures (Fig. 3B). Metastatic mammary tumor cells were identified using the *PyMT*^*+*^*CyclinD1*^*+*^ gene signature (50), whereas general cell types were determined by examining the top 3 to 5 genes per cluster (Supplementary Fig. S5B and S5C; Supplementary Tables S4 and S5). Gene expression analysis identified eight clusters of common cell types (Fig. 3C and D; Supplementary Tables S6 and S7). Significant gene expression changes, associated with high versus low metastatic burden, were observed in dendritic, endothelial, lymphocyte, stromal, monocyte/macrophage, and epithelial cell populations. These data illustrate how dramatically the metastatic microenvironment is altered during metastatic outgrowth.

### Metastatic outgrowth promotes differential gene expression in lung AT2 cells

Lung epithelial cells are the most numerous cells within the lung and are likely to interact extensively with metastatic cells (13). We identified distinct cell types within the epithelial cell cluster using the top 3 to 5 genes per cluster (Fig. 3E). The AT2 cell population, cluster 24, was verified by examining surfactant protein gene expression (Supplementary Fig. S6A and S6B). Of the four lung epithelial cell clusters, AT2 cells showed the most pronounced shift in gene expression in lungs with a high versus low metastatic burden (Fig. 3F). During metastatic outgrowth, AT2 cells upregulated the levels of genes encoding factors involved in wound repair and proliferation and downregulated genes involved in apoptosis and surfactant production (Fig. 3G; Supplementary Tables S8 and S9). This metastasis-associated gene signature is suggestive of an activated AT2 phenotype in which the cells have shifted their functional focus from surfactant production toward a wound repair–related phenotype. Many of these genes are known to contribute to breast cancer and metastatic progression in other cell types (Supplementary Table S9).

### Paracrine interactions with breast cancer cells stimulate AT2 activation

**Figure 4.**
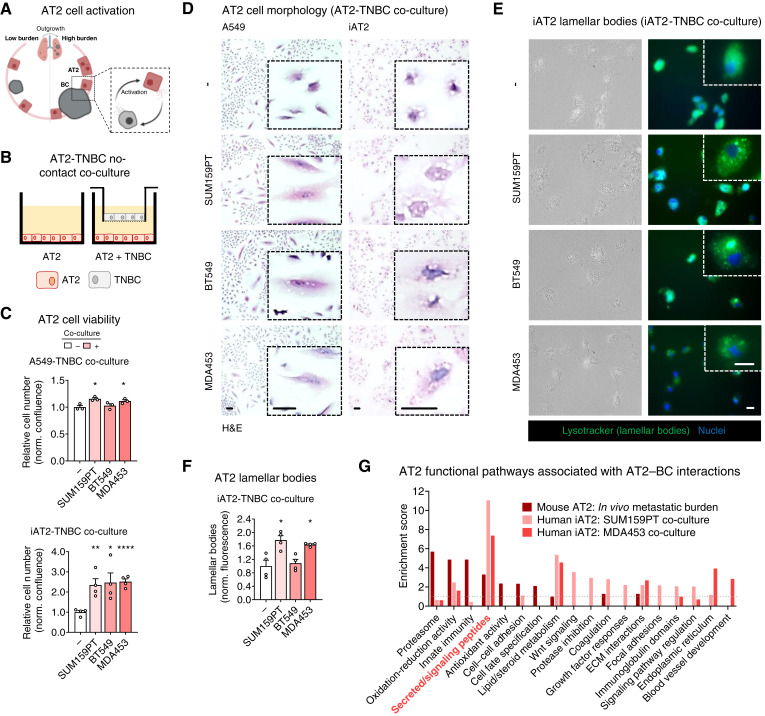
AT2 cell activation by breast cancer (BC) cells. **A,** Model indicating the cellular interactions being interrogated: BC activation of AT2 cells. **B,** Schematic of no-contact co-culture experimental design used to investigate the effects of TNBC cells on lung AT2 cells. **C,** AT2 cell viability was measured by crystal violet assay following co-culture with TNBC cells: 5 days co-culture for A549 cells and 7 days for iAT2 cells. Relative cell numbers were normalized to the mean confluence of AT2 cells cultured alone (−). Mean ± SEM (unpaired *t* tests with Welch correction); *, *P* ≤ 0.05; **, *P* < 0.01; ****, *P* < 0.0001. **D,** AT2 cell morphology was examined by H&E staining following co-culture with TNBC cells for 3 days. Control cells were cultured alone (−). Shown are representative images of cell size and shape; scale bars, 10 μm, inset zoom 4x for A549 cells and 6x for iAT2 cells. **E,** iAT2 lamellar bodies were imaged using lysotracker staining following co-culture with TNBC cells for 3 days. Control cells were cultured alone (−). Shown are representative images of lamellar body size and shape; scale bars, 10 μm, inset zoom 3x. **F,** iAT2 lamellar body levels were measured by quantifying lysotracker staining in iAT2 cells co-cultured with TNBC cells for 3 days. Lamellar body fluorescence was normalized to cell number and the mean fluorescence of iAT2 cells cultured alone (−). Mean ± SEM (unpaired *t* tests with Welch correction); *, *P* ≤ 0.05. **G,** Bulk RNA-seq was performed on RNA collected from iAT2 cells co-cultured with SUM159PT and MDA453 cells for 5 days, whereas control iAT2 cells were cultured alone. Function biological pathways in AT2 cells were determined using DEG lists from mouse AT2 cells in metastatic lungs and human iAT2 cells co-culture with TNBC cells. The enriched pathways from these datasets were then compared to identify overlapping biological pathways. ECM, extracellular matrix.

To directly investigate how breast cancer cells affect AT2 cell activation and function (Fig. 4A), we used three TNBC cells lines (SUM159PT, BT549, and MDA453) and two AT2 cell models (aged A549 cells and differentiated iPSCs). A549 cells are an immortalized lung carcinoma cell line originating from AT2 cells. When cultured long-term (subcultured after thawing more than eight times), A549 cells regain a more AT2 cell–like phenotype with slowed growth and increased expression of AT2-specific surfactant proteins (*SFTP*; Supplementary Fig. S6C; ref. 18). iAT2 cells are created by differentiating iPSC cells through the endoderm stage to AT2 cells (Supplementary Fig. S6D and S6E; ref. 18). Because our *in vivo* data suggest that breast cancer and AT2 cells interact primarily through localized paracrine mechanisms, we used a no-contact co-culture method to study the indirect interactions between these cell types (Fig. 4B). Co-culture with breast cancer cells significantly increased AT2 cell growth compared with AT2 cells cultured alone, as measured by a cell viability assay (Fig. 4C; A549 vs. A549 + SUM159PT *P* = 0.021, A549 vs. A549 + MDA453 *P* = 0.054; iAT2 vs. iAT2 + SUM159PT *P* = 0.019, iAT2 vs. iAT2 + BT549 *P* = 0.053, iAT2 vs. iAT2 + MDA453 *P* = 0.0003). H&E staining of AT2 cells co-cultured with breast cancer cells, when compared with AT2 cells cultured alone, exhibited increased size and an accumulation of intracellular vacuoles (Fig. 4D). Similar changes in cell morphology were observed in AT2 cells treated with CM from SUM159PT cells (Supplementary Fig. S6F). These vacuoles strongly resembled AT2-specific lamellar bodies. To investigate the identity of these vacuoles, iAT2 cells, cultured alone or co-cultured with breast cancer cells, were stained with lysotracker to examine lamellar body levels. The distribution of lamellar body stain was diffuse in iAT2 cells cultured alone, whereas co-culture with TNBC cells caused a more punctate distribution of staining (Fig. 4E). Quantification of this stain showed that co-culture with breast cancer cells promotes lamellar body numbers and/or size in iAT2 cells (Fig. 4F; iAT2 vs. iAT2+SUM159PT *P* = 0.014, iAT2 vs. iAT2 + MDA453 *P* = 0.034). Whether this represents differential surfactant production or changes to surfactant secretion in AT2 cells remains unclear. It does, however, indicate a shift in AT2 cell function from its primary role, facilitating breathing toward a more wound repair–related phenotype.

To better understand how AT2 cells are affected by breast cancer cell-derived secreted factors, we performed bulk RNA-seq on iAT2 cells cultured alone or co-cultured with SUM159PT or MDA453 cells (Supplementary Fig. S6G; Supplementary Table S10). Gene expression in human iAT2 cells (cultured alone, 509 genes) was compared with scRNA-seq data from mouse AT2 cells (low metastatic burden lungs, 194 genes). Little overlap was observed between these two datasets (25 genes; Supplementary Fig. S6H; Supplementary Table S11). Similarly, very little overlap was observed in DEGs in human iAT2 cells (co-cultured with breast cancer cells vs. cultured alone) and those in mouse AT2 cells (high metastatic burden vs. low metastatic burden lungs; 5 genes; Supplementary Fig. S6I; Supplementary Table S11). Functionally, however, several biological pathways overlapped between these data sets, and one pathway in particular, secreted/signaling peptides, was enriched in all three AT2–breast cancer interaction datasets (Fig. 4G; Supplementary Tables S12–S14). These data indicate that paracrine interactions between breast cancer and AT2 cells induce an activated AT2 phenotype characterized by amplified cell growth, altered cell morphology, increased lamellar body numbers, and differential expression of secreted factors (Supplementary Table S15). Functionally, these data suggest that during metastatic outgrowth, AT2 cells may have the ability to dramatically influence the behavior of surrounding cell populations within the lung.

### AT2 secreted factors promote breast cancer cell growth

**Figure 5.**
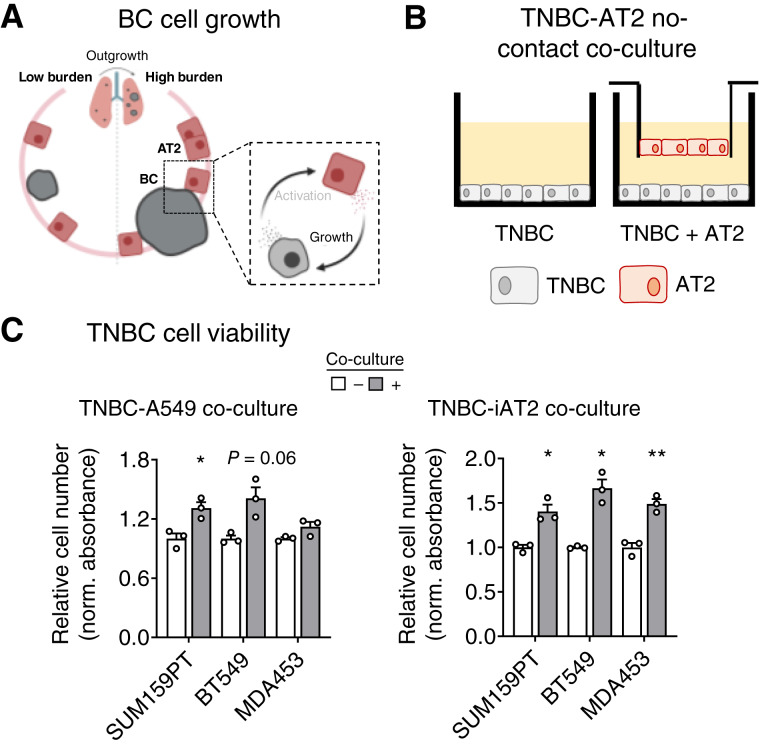
Breast cancer (BC) cell viability in response to AT2 secreted factors. **A,** Model indicating the cellular interactions being interrogated: AT2 induction of BC cell growth. **B,** Schematic of no-contact co-culture experimental design used to investigate the effects of lung AT2 cells on TNBC viability. **C,** TNBC cell viability was measured by crystal violet assay following co-culture with AT2 cells: 5-day co-culture for A549 cells and 7–8 days for iAT2 cells. Relative cell numbers were normalized to the mean absorbance of TNBC cells cultured alone (−). Mean ± SEM (multiple unpaired *t* tests with Welch correction); *, *P* ≤ 0.05; **, *P* < 0.01.

Modified secreted factor gene expression in AT2 cells alludes to the possibility that these cells reciprocally affect breast cancer cell function (Fig. 5A). A complementary no-contact co-culture experiment was performed to investigate the effects of AT2-derived secreted factors on breast cancer cell viability (Fig. 5B). Co-culture with AT2 cells significantly increased TNBC cell growth compared with TNBC cells cultured alone, as measured by a cell viability assay (Fig. 5C; SUM159PT vs. SUM159PT + A549 *P* = 0.019, BT549 vs. BT549-A549 *P* = 0.057; SUM159pT vs. SUM159PT + iAT2 *P* = 0.023, BT549 vs. BT549 + iAT2 *P* = 0.021, MDA453 vs. MDA453 + iAT2 *P* = 0.003). Although the TNBC cell viability FCs were relatively small, as TNBC cell lines have extremely high baseline proliferative rates with doubling times of between 1 to 2.5 days, depending on the cell line tested (51), any increase in proliferation is biologically noteworthy. These data suggest that AT2-derived secreted factors promote breast cancer proliferation within the lung.

### PDE4 inhibition prevents AT2–breast cancer reciprocal activation

**Figure 6.**
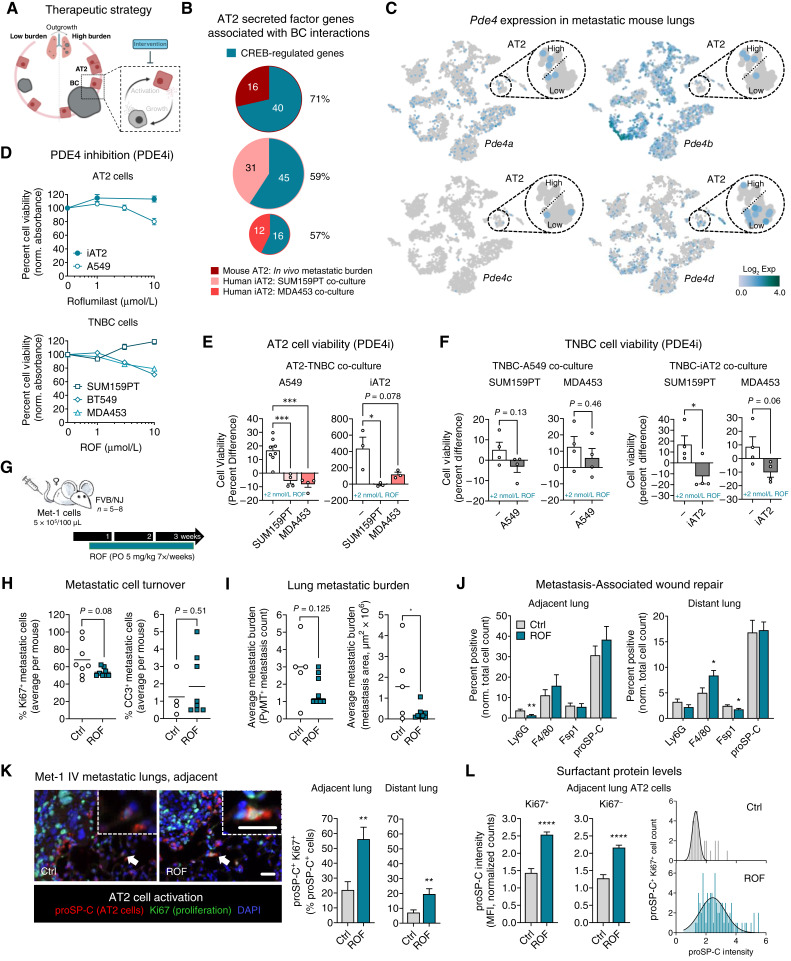
Targeting CREB-regulated AT2 secreted factors to block AT2–breast cancer (BC) interactions and metastatic outgrowth. **A,** Model indicating the cellular interactions being interrogated: targeting AT2–BC interactions during metastatic outgrowth. **B,** The proportion of AT2 secreted factor genes enriched following BC interactions that are CREB-regulated for all three AT2 RNA-seq datasets, percentage indicated. **C,** t-distributed stochastic neighbor embedding visualizations of cells from metastatic mouse lungs clustered by gene expression and colored by *Pde4* isoform gene expression. **D,** Percent cell viability was measured by crystal violet assay after a 3 days of treatment with vehicle DMSO (0) or increasing concentrations of the PDE4 inhibitor (PDEi) ROF. Data were normalized to the mean absorbance of DMSO-treated cells; mean ± SEM. **E,** AT2 cell viability was measured by crystal violet assay following co-culture with TNBC cells and treatment with 2 nmol/L ROF: 5 days for A549 cells and 7 days for iAT2 cells. The percent difference in cell viability, between DMSO and 2 nmol/L ROF treated cells, was calculated for AT2 cells cultured alone (−) or co-culture with TNBC cells. Mean ± SEM (one-way ANOVA with Tukey multiple comparison test); *, *P* ≤ 0.05; ***, *P* < 0.001. **F,** TNBC cell viability was measured by crystal violet assay following co-culture with AT2 cells and treatment with 2 nmol/L ROF: 5 days for A549 cells and 7 days for iAT2 cells. The percent difference in cell viability, between DMSO and 2 nmol/L ROF treated cells, was calculated for TNBC cells cultured alone (−) or co-culture with AT2 cells. Mean ± SEM (unpaired *t* tests); *, *P* ≤ 0.05. **G,** Schematic of metastatic outgrowth experimental design using the late-stage Met-1 metastasis model (*n* = 5–8 mice per group). Met-1 cells were intravenously injected into female mice, and oral (PO) treatment with 5 mg/kg ROF began 3 days later. Mice were treated daily for 3 weeks, and metastatic lung tissue was collected. **H,** Lungs were stained for cell turnover markers Ki67 and CC3 by IHC. The percentage of positively stained cells was scored per metastasis and averaged per mouse; mean (unpaired *t* tests with Welch correction). **I,** Metastatic lungs were stained for the Met-1 mammary-specific marker PyMT. Average metastatic burden was quantified in serial sections as the number of PyMT+ metastases or the size of PyMT+ metastases as measure by area in μm^2^. The average of three serial sections was calculated per mouse. Mean (*n* = 5–8 mice per group, unpaired *t* tests); *, *P* ≤ 0.05. **J,** Metastasis-adjacent and -distant lung tissue was stained for cell-specific markers of wound repair (adjacent: *n* = 16 metastases from five mice per group; distant: *n* = 64 regions of interest from 5–8 mice per group). The number of positively stained cells, normalized to the total number of cells, was quantified in the 300 μm surrounding metastases for the adjacent lung and the full region of interest in the distant lung. Mean ± SEM (multiple unpaired *t* tests with Welch correction); *, *P* ≤ 0.05; **, *P* < 0.01. **K,** Lungs adjacent and distant to large metastases were stained for AT2 cell activation. Shown are representative images of large metastases from control (Ctrl) and ROF-treated mice indicating proSP-C and Ki67 double-positive proliferating AT2 cells (white arrows); scale bar, 5 μm, inset zoom 3x. The percentage of proSP-C–positive cells that are Ki67-positive was quantified in the lung (adjacent: *n* = 21 metastases from 3–4 mice per group; distant: *n* = 50 regions of interest from 4–6 mice per group). Mean ± SEM (unpaired *t* tests with Welch correction); **, *P* < 0.01. **L,** The mean fluorescent intensity (MFI) of proSP-C in metastasis-adjacent proSP-C^+^ AT2 cells was quantified in Ki67^+^ and Ki67^−^ cells (*n* = 21 metastases from 3–4 mice per group). Mean ± SEM (*t* tests with Welch correction); ****, *P* < 0.0001. Shown are representative distributions demonstrating the number of cells per mouse expressing increasing levels of proSP-C.

Overall, our data indicate that throughout metastatic outgrowth and the development of metastasis-associated wound repair, AT2 cells become activated in the presence of breast cancer cells and, subsequently, AT2 cells reciprocally promote breast cancer cell growth through indirect paracrine signaling. We hypothesized that interrupting the reciprocal paracrine interaction between AT2 and breast cancer cells may inhibit lung metastatic outgrowth (Fig. 6A). To identify a suitable target for pharmaceutical intervention, we examined predicted upstream regulators of differentially expressed secreted factors identified in AT2 cells from high–metastatic burden mouse lungs and TNBC co-cultures. We focused primarily on transcriptional regulators with established treatment strategies that could easily be repurposed for use in patients with breast cancer. cAMP CREB was identified as a potential upstream regulator of AT2 secreted factor genes in all three datasets by IPA (mouse metastatic lungs *P* = 9.77E−05, SUM159PT co-culture *P* = 3.93E−03, and MDA453 co-culture *P* = 3.12E−03; ref. 29). Notably, using a gene signature developed by Zheng and colleagues (26) in which they knocked down and rescued CREB in cancer cells to identify CREB-regulated genes, we found that a large percentage of breast cancer–induced AT2 secreted factors, identified in RNA-seq datasets from both human and mouse cells, are potentially CREB-regulated (Fig. 6B; Supplementary Fig. S7A; Supplementary Table S15).

CREB is the transcription factor responsible for regulation of genes associated with the cAMP–protein kinase A (PKA)–CREB signaling axis. cAMP–PKA–CREB signaling regulates a number of physiologic processes, and aberrant signaling plays a role in a variety of cancers (52). cAMP has been specifically linked to cytokine secretion in lung epithelial cells (53), and CREB is considered to be a key regulator of breast cancer metastasis (54). cAMP–PKA–CREB signaling, which has an overall anti-inflammatory effect, is negatively regulated by PDE activity through the hydrolysis of cAMP (55). PDE4 is one of several PDEs in the lung, where it is ubiquitously expressed and collectively contributes to organ-wide inflammatory responses (56). Notably, PDE4 is the primary PDE expressed in AT2 cells (57). In normal, healthy lung tissue and AT2 cells from humans and mice, *PDE4* isoforms (*PDE4A-D*) are expressed at relatively low levels (Supplementary Fig. S7B; ref. 58). In mouse lungs with metastases, *Pde4* isoforms are expressed at various levels in many cell types (Fig. 6C). During metastatic outgrowth, isoform expression in AT2 cells seems to shift from *Pde4c* and *Pde4d* in lungs with a low metastatic burden toward *Pde4a* and *Pde4b* in lungs with a high metastatic burden, though these changes are observable trends and not statistically significant (Fig. 6C).

Nonsteroidal anti-inflammatory PDE4 inhibitors are commonly used to treat pathologic lung conditions. ROF is a routinely used second-generation oral PDE4 inhibitor FDA-approved for the treatment of COPD. Dose–response cell viability experiments were performed to test the sensitivity of AT2 and TNBC cells to increasing doses of ROF. Although some statistical differences were observed with varying doses of ROF, none of the data illustrated biologically relevant effects on cell viability in AT2 or TNBC cells (cell viability following 10 μmol/L ROF ranging from 71%–119%; Fig. 6D), especially considering physiologically relevant doses of ROF are between 1 and 2 nmol/L (59). Similar results were observed in ER+ breast cancer cells treated with ROF (Supplementary Fig. S7C). Likewise, the sensitivity of AT2 and TNBC cell viability was examined in response to increasing doses of another second-generation PDE4 inhibitor cilomilast (CILO), and no biologically relevant effects on cell viability were observed (Supplementary Fig. S7D). Met-1 mouse mammary carcinoma cells also showed little response to PDE4 inhibition, consistent with what we observed in human breast cancer cells (Supplementary Fig. S7E). These data indicate that AT2 and breast cancer cells are resistant to ROF when cultured alone.

To test whether breast cancer–induced activation sensitizes AT2 cells to PDE4 inhibition, AT2 cell viability was examined following simultaneous treatment with CM from TNBC cells and ROF. A549 cells were sensitized to ROF when activated by secreted factors from TNBC cells, as measured by reduced cell viability compared with A549 cells cultured in normal media (Supplementary Fig. S7F). Similarly, as the data indicated that AT2 and TNBC cells reciprocally promote growth when cultured in no-contact co-culture conditions (Figs. 4C and 5C), we tested whether reciprocal activation may be necessary to sensitize AT2 and TNBC cells to ROF. To investigate this, AT2 and TNBC cell viability was quantified following 2 nmol/L ROF treatment with or without co-culture. Surprisingly, when cultured alone in a physiologically relevant doses of 2 nmol/L ROF, cell growth was marginally increased in several cell lines (Supplementary Fig. S7G), but AT2-TNBC paracrine interactions reversed these ROF effects. ROF inhibited cell viability in AT2 cells co-cultured with TNBC cells (Fig. 6E; A549 vs. A549 + SUM159PT *P* = 0.0006, A549 vs. A549 + MDA453 *P* = 0.0002; iAT2 vs. iAT2 + SUM159PT *P* = 0.021). Similarly, TNBC cell viability was inhibited by ROF only when co-cultured with AT2 cells (Fig. 6F; SUM159PT vs. SUM159PT + iAT2 *P* = 0.033). These data suggest that reciprocally activated AT2-TNBC cells respond favorably to PDE4 inhibition.

### PDE4 blockade limits lung metastatic outgrowth

PDE4 inhibition, using ROF, as a treatment for patients with breast cancer with lung metastases is a promising therapeutic strategy. Not only do our data suggest that ROF impedes the progrowth effects of AT2 cells on breast cancer cells during metastasis, but it may also abrogate the chronic wound repair that develops during metastatic outgrowth. Thus, we tested the effectiveness of ROF in blocking metastatic outgrowth using a late-stage metastasis mouse model of breast cancer. Once Met-1 cells colonized the lungs, 3 days following tail vein injection (60–62), mice were administered oral ROF daily at a dose of 5 mg/kg for 3 weeks (Fig. 6G). Daily treatment with ROF was well-tolerated, with no changes in mouse weight over the course of the experiment (Supplementary Fig. S7H).

Analysis of whole-lung lysates showed that several known CREB target genes were downregulated following ROF treatment (Supplementary Fig. S7I), indicating successful targeting of CREB signaling via PDE4 inhibition throughout the lung. To examine the tumor cell response to ROF, we stained metastatic lungs by IHC for proliferation and cell death markers, Ki67 and CC3, respectively. Although the data did not reach statistical significance, there is a trend toward reduced metastatic proliferation in ROF-treated lungs with little to no change in cell death (Fig. 6H; Supplementary Fig. S7J). Metastatic burden was analyzed by IHC staining lung serial sections for the Met-1 mammary-specific marker PyMT. Whereas ROF treatment had no effect on the number of metastases, it significantly reduced the size of lung metastases (Fig. 6I; Ctrl vs. ROF *P* = 0.045 and Supplementary Fig. S7K) suggesting that ROF inhibited outgrowth of established lung micrometastases rather than killing metastatic cells.

To test how ROF affects metastasis-associated wound repair, mouse lungs were stained using our multi-IF wound repair panel. In the lungs directly adjacent to large metastases, ROF treatment caused a decrease in Ly6G^+^ neutrophils (*P* = 0.006), whereas there was an increase in F4/80^+^ macrophages (*P* = 0.017) accompanied by a decrease in Fsp1^+^ fibroblast (*P* = 0.020) in the distant lung (Fig. 6J). These data indicate that the ROF alters wound repair/immune responses in metastatic lungs and that these effects may be unique depending on the proximity of immune cells to metastatic cells. Interestingly, the number of AT2 cells adjacent to metastases was unchanged following ROF treatment (Fig. 6J). To investigate whether AT2 activation was altered by ROF treatment, mouse lungs were co-stained for the AT2-specific marker proSP-C and Ki67. Significantly more proliferating AT2 cells were present in the lung adjacent to large metastases in ROF-treated mice compared with control-treated mice (*P* = 0.004). Similarly, an increased number of proliferating AT2 cells were present in the distant lung (Fig. 6K; *P* = 0.003). These data suggest that ROF induces hyperactivation of AT2 cells within the lung. Visualization of this staining also suggested that ROF treatment may alter proSP-C expression in AT2 cells. Thus, the intensity of proSP-C staining was examined in AT2 cells adjacent to metastases. There was an obvious shift toward increased proSP-C expression in AT2 cells following ROF treatment, regardless of their proliferative status (Fig. 6L; Ki67^+^*P* < 0.0001, Ki67^−^*P* < 0.0001), suggesting a concomitant shift in phenotype and function that may correspond to a more antimetastatic phenotype, though further investigation is necessary to fully characterize these effects. Overall though, our preclinical analysis suggests that PDE4 inhibition directly targets AT2 cell activation and may be a viable therapeutic strategy in patients with breast cancer with lung metastasis.

### PDE4 is elevated in the lungs of patients with metastatic breast cancer

**Figure 7.**
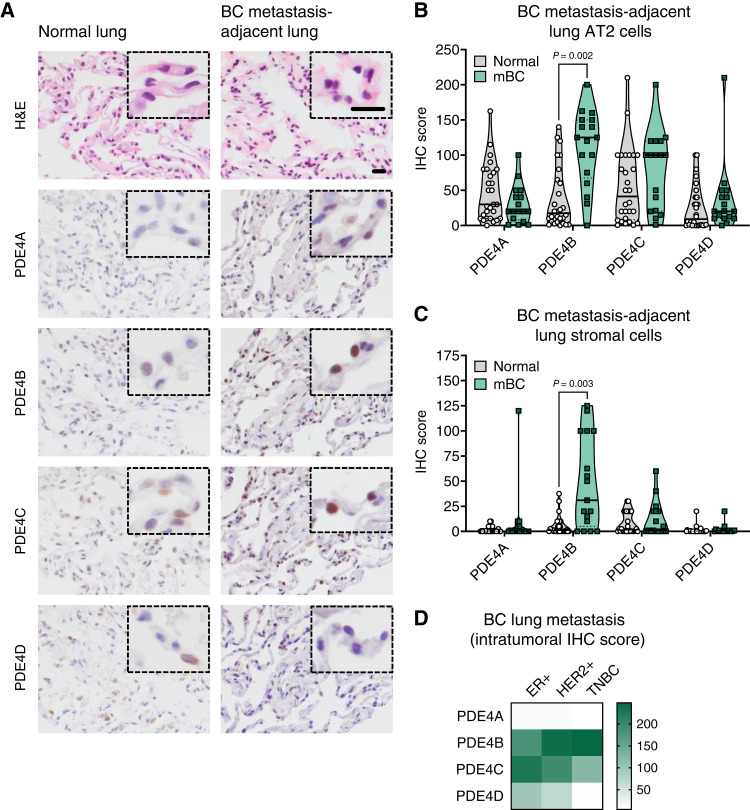
PDE4 isoform levels in lungs from patients with breast cancer (BC) with metastases. **A,** Lungs from normal (*n* = 28) and patients with BC with metastases (*n* = 17; Supplementary Table S1C for specimen demographics) were IHC-stained for PDE4 isoforms A–D. Shown are representative images from normal and metastasis-adjacent human lungs; scale bar, 20 μm, inset zoom 3x. **B–D,** Protein expression was scored by a clinical pathologist and presented as an IHC score. Isoform levels were scored in lung AT2 cells (**B**), the lung stroma, which included all metastasis-adjacent cells except AT2 cells (**C**), and metastases (**D**). Median and quartiles are indicated on each graph (nonparametric Kolmogorov–Smirnov tests for grouped data and nonparametric Kruskal–Wallis test for the intratumoral staining comparison). mBC, metastatic breast cancer.

Based on our data from mice and the development of chronic wound repair in the lung during metastatic outgrowth, we hypothesized that PDE4 levels would be increased in lungs from breast cancer patients with metastases when compared with normal lungs. To examine this, protein expression of PDE4 isoforms A-D was quantitatively scored by a pathologist in lungs from patients with metastatic breast cancer and compared with normal lungs collected during autopsy or following traumatic injury (Fig. 7A). As predicted by publicly available scRNA-seq analyses (Supplementary Fig. S7B), human lungs express relatively low protein levels of all PDE4 isoforms, regardless of sex or cell type (Supplementary Fig. S8A and S8B). Similarly, no significant differences were observed in PDE4 levels by age in normal lungs from women, regardless of cell type (Supplementary Fig. S8C). Future analysis of larger datasets will be required to definitively determine how PDE4 expression in the lung may change during aging. Importantly, the main source of PDE4 in human lungs is AT2 cells (Supplementary Fig. S8A–S8C). This holds true in lungs from women with metastatic breast cancer, and PDE4B levels in AT2 cells from metastatic lungs are significantly higher than those from normal lungs (Fig. 7B; Supplementary Fig. S8D). A similar effect is observed in stromal cells within the lung adjacent to metastases, with PDE4 being most frequently expressed in fibroblasts and lymphocytes (Fig. 7C; Supplementary Fig. S8D). No significant differences were observed in metastatic intratumoral PDE4 levels depending on breast cancer subtype, but our data may indicate that relative PDE4 isoform levels vary depending on the molecular signature (Fig. 7D; Supplementary Fig. S8E). Taken together, these data suggest that metastasis-adjacent AT2 cells upregulate PDE4B during metastatic outgrowth and supports our hypothesis that PDE4 may serve as an effective therapeutic target in patients with metastatic breast cancer.

## Discussion

When defining the stages of the metastatic cascade, many studies and reviews end the cascade at the stage of colonization. Outgrowth is a phase that is often overlooked as a distinct stage of metastasis and understudied in preclinical models. However, outgrowth of metastatic disease significantly affects patient survival and quality of life. Our study shows that widespread cell-specific changes occur during metastatic outgrowth within the lung (Fig. 3). During outgrowth, unique, critical interactions between cancer cells and resident cells within the metastatic tissue occur that support progression to overt metastasis. This study identified targetable reciprocal interactions that may lead to treatment strategies for patients with breast cancer lung metastases.

Growing metastases cause damage to surrounding tissue within the lung, leading to a chronic wound repair response. Interestingly, our data indicates that although there are many similarities to classic wound repair, metastasis-associated wound repair is a unique process likely caused by a combination of physical injury and cancer cell paracrine signaling (Figs. 1 and 2). AT2 cell function, in particular, is significantly altered during metastatic outgrowth (Figs. 3 and 4). Our data are consistent with previous studies noting the potential importance of AT2 cells in premetastatic niche formation and metastatic colonization.

The Malanchi group found that a stem-like progenitor phenotype was activated in AT2 cells adjacent to early metastases (16, 17). This fits with the stem cell role of AT2 cells in the alveolar niche during injury and overlaps with our data suggesting that AT2 cells are adopting a wound repair–related phenotype during metastatic outgrowth. However, although there were some similarities between our data and gene signatures developed by Malanchi, there were significant differences as well. We also examined whether metastasis-adjacent AT2 cells were merely acquiring a damage-associated tissue progenitor (DATP) activation state that is commonly observed in AT2 cells following alveolar injury (63–65). Our scRNA-seq data indicate that *Il1r1* expression, which is an important initiator of DATP (63), in AT2 cells trends down with metastatic outgrowth in the mouse lung. In fact, there was only limited overlap between a more extensive established DATP gene signature (66) and genes induced in mouse and human AT2 cells following interactions with breast cancer cells. These comparisons support our hypothesis that AT2 cells develop a unique activation signature during metastatic outgrowth that subsequently supports metastatic progression (Figs. 5 and 6).

Our studies identified a targetable mechanism that can be modulated in the clinic with an FDA-approved drug. Several functional biological pathways were altered in AT2 cells following interaction with breast cancer cells, and our investigation focused on the commonly enriched secreted/signaling peptides pathway as a potential mechanism for how AT2 cells promote TNBC cell viability. We identified several CREB-regulated AT2 secreted factors involved in inflammation, immune response, and chemotaxis, which could dramatically affect cell behavior within the surrounding microenvironment (Supplementary Table S14). PDE4 inhibition in lung epithelial cells reduces inflammatory/immune secreted factor levels and has previously been shown to enhance resolution of inflammation in the lung (67). By inhibiting PDE4 activity, we successfully reduced reciprocal AT2–breast cancer cell activation and metastatic outgrowth (Fig. 6). Importantly, AT2 cells play a critical role in regulating immune response within the alveolus through integration of multi-cellular signals. Thus, changes to AT2 gene expression and function could cause a cascade of effects within the lung microenvironment with a collective antimetastatic effect. This effect might also be complemented by the anti-inflammatory consequences of PDE4 inhibition on other cells within the lung, where PDE4 is ubiquitously expressed (56). Importantly, an investigation of PDE4 isoform expression in human lungs from patients with breast cancer with metastases showed that AT2 cells are the main expressors of PDE4B and that PDE4B levels are elevated in the lungs of patients with metastases when compared with normal lungs (Fig. 7). Thus, our preclinical *in vitro* and *in vivo* data seems to reflect what happens during metastatic outgrowth in patients with breast cancer.

Several PDE4 inhibitors are FDA-approved to treat inflammatory conditions, but ROF is the only PDE4 inhibitor currently approved for the treatment of COPD. For over a decade, patients with COPD have been prescribed a daily oral dose of 500 μg ROF to counter the long-term inflammatory consequences of COPD (59). ROF is generally well-tolerated (68), and the safety of extended daily treatment is firmly established (69). There is some evidence suggesting that ROF may be associated with an increased incidence of certain cancers (70), but other clinical studies have shown its effectiveness as an anti-cancer therapy, particularly when given in combination with chemotherapy (71, 72). Our data strongly suggest that ROF may serve as a viable lung metastasis–specific treatment strategy, but future studies will investigate its overall antitumor effects in breast cancer, particularly when combined with standard-of-care chemotherapy. Future studies will also investigate its effects on the early stages of metastasis, given the importance of AT2 cells in premetastatic niche preparation and metastatic colonization, as well as its effects on different breast cancer subtypes and later stages of metastasis such as secondary metastatic spread to organs beyond the lung. Finally, as ROF caused little-to-no cell death of metastases, future studies need to determine whether its growth inhibitory effects cease when drug treatment is discontinued. Determining whether ROF induces irreversible cellular senescence or reversible quiescence/dormancy will help to pinpoint which anticancer drugs will be most effective when combined with ROF.

PDE4 inhibitor development is proceeding at a rapid pace. ROF is a nonspecific PDE4 inhibitor that blocks activity of all PDE4 subtypes with a high affinity for PDE4B and PDE4D. Our interrogation of publicly available scRNA-seq data (Supplementary Fig. S7B) and metastatic patients with breast cancer (Fig. 7) indicated that *PDE4* isoform levels are context-dependent, and our data indicate that expression may vary depending on the stage of disease progression. Isoform-specific inhibitors are under development and may prove to be more effective against lung metastases. Additionally, inhaled PDE4 inhibitor therapy is being investigated to directly target inflammation in the lung (73). The FDA recently approved nebulizer delivered ensifentrine, a dual PDE3/PDE4 inhibitor, for the treatment of adults with COPD (74). As many of the effects observed in our studies are localized to the lung directly adjacent to metastases, inhaled administration of drugs may be a better way to target focal microenvironmental changes induced by growing metastases. Future studies are needed to clarify the most efficacious mode of PDE4 targeted therapeutic intervention for patients with lung metastases. Ultimately, this study indicates that targeting metastasis-associated wound repair and AT2 activation during lung metastatic outgrowth may prove to be a promising avenue for the treatment of metastatic breast cancer.

## Supplementary Material

Supplementary Methods Materials and Methods

Supplementary Figure 1 Histological analysis of lung metastases.

Supplementary Figure 2 Multispectral immunofluorescent analysis of lung metastases.

Supplementary Figure 3 Cell activation in metastatic lungs.

Supplementary Figure 4 scRNAseq dissociation gene signature analysis.

Supplementary Figure 5 scRNAseq analysis of metastatic outgrowth in lungs.

Supplementary Figure 6 Lung AT2 cells.

Supplementary Figure 7 PDE4 levels and inhibition.

Supplementary Figure 8 PDE4 isoform levels in human lungs.

Supplementary Table 1 Methods.

Supplementary Table 2 Lung scRNAseq Bulk comparison: Low vs High.

Supplementary Table 3 Lung scRNAseq Bulk comparison: Low vs High - Pathways.

Supplementary Table 4 Lung scRNAseq cell clusters.

Supplementary Table 5 Lung scRNAseq cell clusters: Low vs High.

Supplementary Table 6 Lung scRNAseq cell types.

Supplementary Table 7 Lung scRNAseq cell types: Low vs High.

Supplementary Table 8 Lung scRNAseq AT2 cells: Low vs High.

Supplementary Table 9 AT2 top gene summary.

Supplementary Table 10 iAT2 cells co-cultured with TNBC cells.

Supplementary Table 11 AT2 dataset gene overlap.

Supplementary Table 12 AT2 pathways associated with BC interaction.

Supplementary Table 13 AT2 pathway clusters.

Supplementary Table 14 AT2 secreted factor gene groups.

Supplementary Table 15 AT2 secreted factor genes.

## Data Availability

Data, methods, and resources supporting the findings of this study which are not available in the manuscript or supplementary materials are available from the corresponding author upon request. RNA-seq gene expression data are publicly available through the GEO database (mouse lung scRNA-seq: GSE319843, iAT2-BC co-culture bulk RNA-seq: GSE294453).
