# Supplementary material for: Metastasis-Associated Wound Repair Promotes Reciprocal Lung Epithelium Activation and Breast Cancer Metastatic Outgrowth
Source: Cancer Res Commun. 2026 Apr 6;6(4):750–68. doi: 10.1158/2767-9764.CRC-25-0459 (PMC13051055; doi:10.1158/2767-9764.CRC-25-0459)
Supplement: Supplementary Methods — Materials and Methods [file crc-25-0459_supplementary_methods_suppsm.pdf]

SUPPLEMENTARY MATERIALS AND METHODS

In vivo mouse experiments.

Lung MRI scans. Each mpMRI session consisted of a tri-pilot localizer, followed by gated fast spin echo proton density weighted PD-RARE (Rapid Acquisition with Refocusing Echoes) sequences in axial and coronal planes using a 38-mm mouse body RF coil. The following sequence parameters were used: field of view 30x30 mm; slice thickness 1 mm; number of slices 20 (coronal) and 24 (axial); repetition time TR=2200 ms; echo time TE=32 ms; RARE factor 8; number of averages 4; matrix size 320x320. Total acquisition time for all sequences was 11 min 6 sec. MRI image acquisition and analysis was performed using Bruker ParaVision 360neo v2.0 software by a MRI physicist with >15 years of experience.

Histology.

IHC Metastasis size. Size was determined using ImageJ software. Each metastasis was circled to compute area in pixels^2. Metastasis diameter in µm was calculated using the conversion factor 1.25 ImageJ pixels = 1 µm and the following calculations:

$$\text{metastasis area } (\mu m^2) = \left(\frac{\sqrt{\text{metastasis area in pixels}^2}}{1.25}\right)^2$$

$$\text{metastasis diameter } (\mu m) = \left(\sqrt{\frac{\text{metastasis area}}{\pi}}\right) \times 2$$

When comparing small, medium and large metastases: small metastases had a diameter (d) <150µm, medium metastases d=150-300µm, and large metastases d>300µm. When comparing small versus large metastases: small metastases had d<200µm and large metastases d>200µm. ImageJ was also used to quantify IHC staining in the adjacent 100µm of lung surrounding metastases (Supplementary Figure 1A). Data was presented as the percentage of positively stained cells relative to the total number of cells.

Lung compaction investigation. This analysis compared cellular content in the same total area of the lung surrounding metastases regardless of metastasis size (excluding metastatic and intratumoral cells). The 100µm surrounding the largest lung metastasis in the MMTV-PyMT lung cohort, with a radius of 325µm, was used to determine the total area being compared surrounding all metastases, ~5.7×10^5 µm^2 (radius of 425µm from the center of each metastasis). ImageJ was used to quantify cell counts in the lung surrounding metastases. The cellular count from our smallest metastases, with a radius of 25µm, was used as a reference for comparison. Data was presented as the number of cells normalized to the cell count for the smallest metastasis (Supplementary Figure 1E).

Multispectral immunofluorescence (multi-IF). Only ~1-2% of each image included metastatic cells; the remaining image area was focused on surrounding lung tissue and vasculature, ~96% and ~4% respectively (Supplementary Figure 1B). Metastasis size was determined using Phenochart software (Akoya Biosciences). The width and height (μm) of each metastasis was measured and averaged to calculate metastasis diameter. When comparing small versus large metastases: small metastases had d<200μm and large metastases d>200μm.

Metastatic microenvironment analysis. Basic mathematical equations for a circle (listed below) were used in multi-IF analyses to determine: 1) the area adjacent to metastases, within a radius of ≤300μm, and 2) whether individual cells were located adjacent or non-adjacent to metastases (Supplementary Figure 1B). A comprehensive wound repair score was calculated by combining the counts for all cell types (neutrophils, macrophages, fibroblasts, and AT2 cells) within the lung adjacent to metastases. Data are presented as the number of positive cells normalized to area.

$$A = \pi r^2$$

$$d = r \times 2$$

A = area

r = radius

d = diameter

$$r^2 = (x - h)^2 + (y - k)^2$$

(h, k) = location of the center of the circle (corner of the ROI with the metastasis)

(x, y) = location of the cell of interest

Bioinformatics

Enzymatic dissociation of lungs for scRNAseq. We developed a protocol for enzymatic dissociation of fresh mouse lungs based on multiple published techniques [1-3]. Mice were perfused with 10mL 1X Hank's balanced salt solution (HBSS; Corning CellGro, No. 21-023-CV). Lungs were collected and rinsed in chilled 1X PBS. The tissue was cut into small pieces with a razor blade and incubated in 5mg/mL type 2 collagenase (Worthington Biochemical Corporation, No. LS004174) in phenol-red free DMEM for 10min at 37ºC on a benchtop shaker. Enzyme A/Buffer Y mix from the Neural Tissue Dissociation Kit P from Miltenyi Biotec (No. 130-092-628) was added to the collagenase-tissue mixture and incubated for another 15min at 37ºC on a benchtop shaker. Pipetting periodically during this incubation was used to dissociate the tissue. Ice cold PBS/bovine serum albumin (BSA) cell buffer (1X PBS with 0.5% 50mg/mL BSA; ThermoFisher Scientific, No. AM26160) was used to halt the dissociation enzymatic reaction. Cells were passed through a 100μm mesh filter and centrifuged at 300 xg for 5min at 4ºC. Cells were then resuspended in ammonium-chloride-potassium (ACK) buffer for 1min at 20ºC to lyse any remaining red blood cells. Cold PBS/BSA cell

buffer was added and cells centrifuged at 300 xg for 10min at 4ºC. Cells were resuspended in cold 1X PBS and debris was removed using cold Debris Removal Solution (Miltenyi Biotech, No. 130-109-398) according to the manufacturer's instructions. Cells were washed again with PBS/BSA cell buffer and centrifuged at 300 xg for 5min at 4ºC. Finally, cells were resuspended in 500µL-1mL PBS/BSA cell buffer and filtered through a 40µm Flowmi cell strainer (Millipore Sigma, No. BAH136800040).

References

1. Yamazaki A, Shue F, Yamazaki Y, Martens YA, Bu G, Liu CC: Preparation of single cell suspensions enriched in mouse brain vascular cells for single-cell RNA sequencing. *STAR Protoc* **2021**;2(3):100715.
2. Lancelin W, Guerrero-Plata A: Isolation of mouse lung dendritic cells. *J Vis Exp* **2011**;(57).
3. Lambrechts D, Wauters E, Boeckx B, Aibar S, Nittner D, Burton O *et al.*: Phenotype molding of stromal cells in the lung tumor microenvironment. *Nature Medicine* **2018**;24(8):1277-89.
