# Supplementary material for: Metastasis-Associated Wound Repair Promotes Reciprocal Lung Epithelium Activation and Breast Cancer Metastatic Outgrowth
Source: Cancer Res Commun. 2026 Apr 6;6(4):750–68. doi: 10.1158/2767-9764.CRC-25-0459 (PMC13051055; doi:10.1158/2767-9764.CRC-25-0459)
Supplement: Supplementary Figure 1 — Histological analysis of lung metastases. [file crc-25-0459_supplementary_figure_1_suppsf1.pdf]

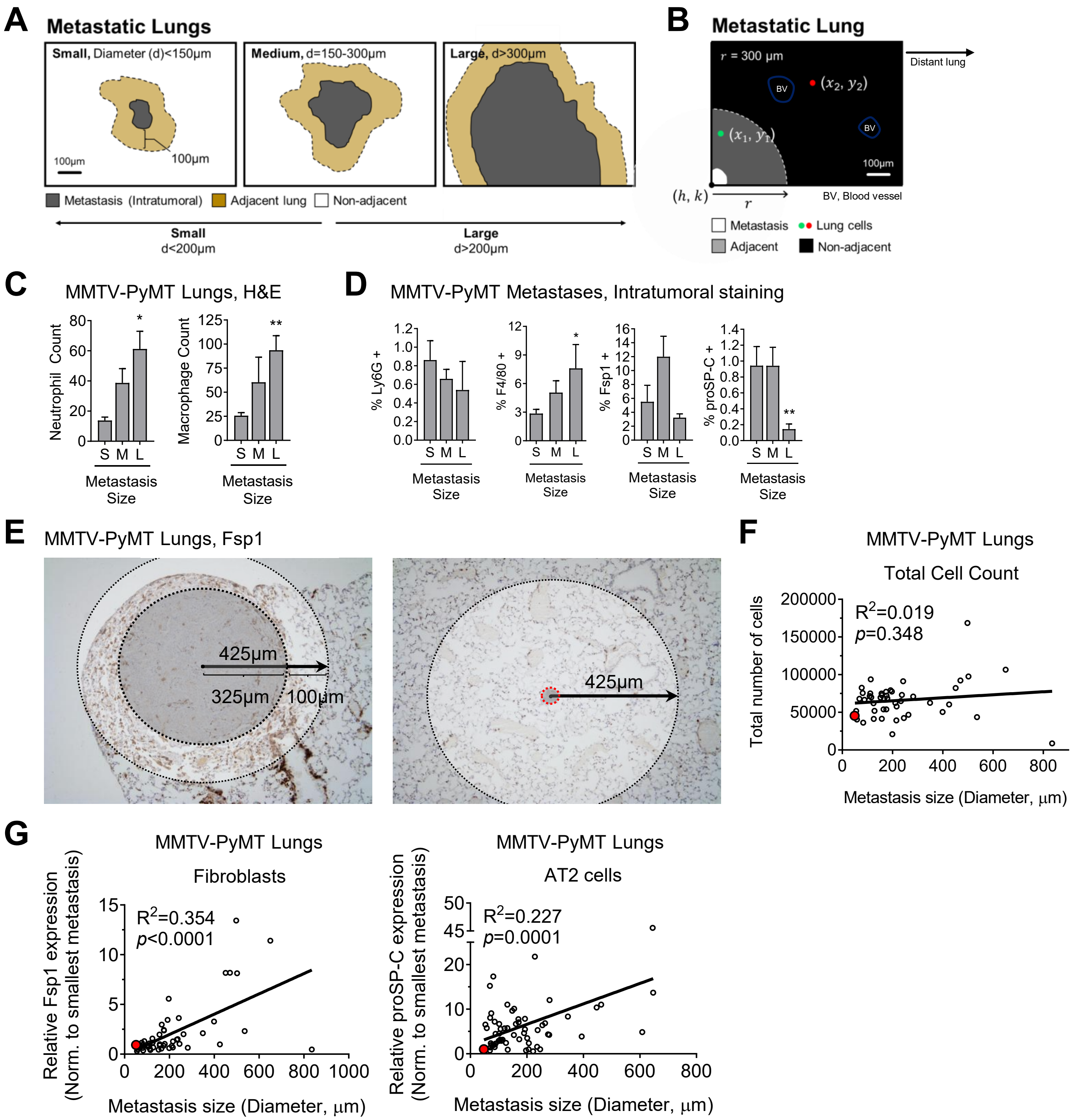

Supplementary Figure 1.

Supplementary Figure 1. Histological analysis of lung metastases. A, Model defining metastasis size designations based on diameter in µm and classifying metastasis adjacent versus non-adjacent tissue in the lung microenvironment for IHC stains. **B,** Model depicting how multispectral immunofluorescence data was quantified and how cells were determined to be metastasis adjacent versus non-adjacent versus distant. **C,** MMTV-PyMT metastatic lungs were H&E stained (*n*=20 metastases from 3-4 mice per stain; see Supplementary Table 1 for sample number details). The number of neutrophils and macrophages adjacent to metastases were counted by a veterinary pathologist. Mean ± SEM (unpaired *t*-tests with Welch's correction); * $p \leq 0.05$, ** $p < 0.01$. S, small; M, medium; L, large metastases. **D,** MMTV-PyMT metastatic lungs were stained for cell-specific markers of lung wound repair ($n > 50$ metastases from 2-7 mice per stain). The percentage of positively stained intratumoral metastatic cells, normalized to the total number of cells, was quantified. Mean ± SEM (unpaired *t*-tests with Welch's correction); * $p \leq 0.05$, ** $p < 0.01$. S, small; M, medium; L, large metastases. **E,** Images depicting how lung compaction surrounding growing metastases was investigated in MMTV-PyMT metastatic lungs stained by IHC for the fibroblast marker Fsp1 and the AT2 marker proSP-C. The 425µm surrounding each metastasis (selected by measuring the 100µm surrounding the largest lung metastases) was analyzed for positively stained cells. **F,** The number of cells surrounding metastases in MMTV-PyMT lungs was quantified in lungs stained for Fsp1. Total number of cells relative to metastasis size for each metastasis was quantified. The red circle represents data for the smallest metastasis within our dataset (linear regression analysis from n>49 metastases in 7 mice). **G,** The number of positively stained cells surrounding metastases in MMTV-PyMT lungs was normalized to the data from the smallest metastasis (red circle) and correlated to metastasis size (linear regression analysis from n>49 metastases in 7 mice).
