# Supplementary material for: Metastasis-Associated Wound Repair Promotes Reciprocal Lung Epithelium Activation and Breast Cancer Metastatic Outgrowth
Source: Cancer Res Commun. 2026 Apr 6;6(4):750–68. doi: 10.1158/2767-9764.CRC-25-0459 (PMC13051055; doi:10.1158/2767-9764.CRC-25-0459)
Supplement: Supplementary Figure 2 — Multispectral immunofluorescent analysis of lung metastases. [file crc-25-0459_supplementary_figure_2_suppsf2.pdf]

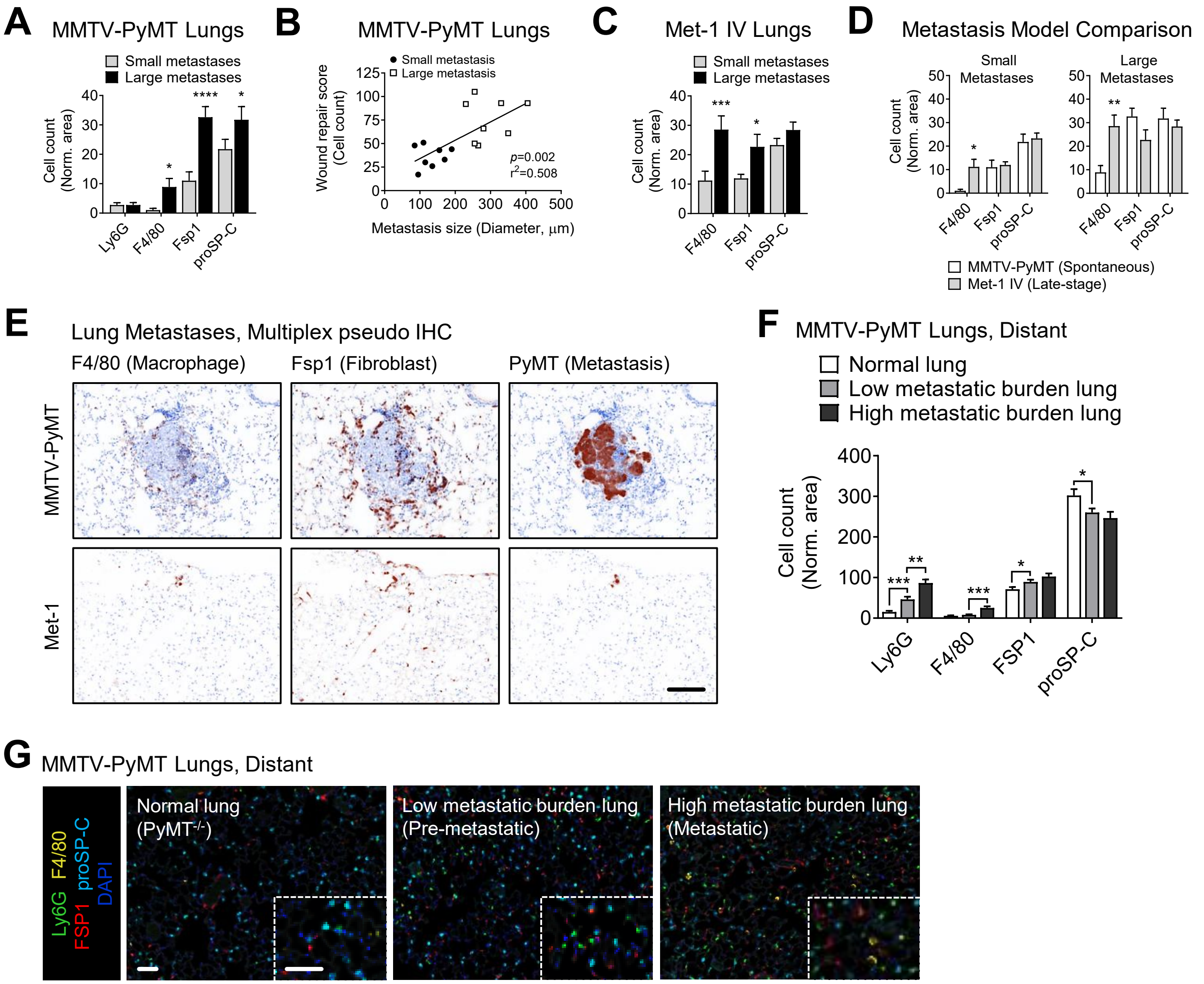

Supplementary Figure 2.

Supplementary Figure 2. Multispectral immunofluorescent analysis of lung metastases. A, MMTV-PyMT metastatic lungs were stained for cell-specific markers of lung wound repair (*n*=16 metastases from 2-4 mice per group). The number of positively stained cells, normalized to area, was quantified in the 300µm surrounding metastases. Mean ± SEM (multiple unpaired *t*-tests); * *p*≤0.05, **** *p*<0.0001. **B,** Wound repair scores from MMTV-PyMT lungs relative to metastasis size (linear regression from *n*=16 metastases in 2-4 mice). **C,** Met-1 metastatic lungs were stained for cell-specific markers of lung wound repair (*n*=50 metastases from 5 mice). The number of positively stained cells, normalized to area, was quantified in the 300µm surrounding metastases. Mean ± SEM (multiple unpaired *t*-tests); * *p*≤0.05, *** *p*<0.001. **D,** The number of positively stained cells surrounding metastases, normalized to area, was compared in the spontaneous MMTV-PyMT metastasis model (*n*=16 metastases from 2-4 mice) and the late-stage Met-1 IV metastasis model (*n*=50 metastases from 5 mice). Mean ± SEM (multiple unpaired *t*-tests); * *p*≤0.05, ** *p*<0.01. **E,** Lungs from MMTV-PyMT and Met-1 metastasis models were stained by multispectral immunofluorescence for cell-specific markers of wound repair. Shown are representative pseudo-colored images; scale bar = 25µm. **F-G,** Lungs from wild-type mice ($PyMT^{-/-}$) and MMTV-PyMT mice with low (pre-metastatic) or high metastatic (metastatic) burden were stained for cell-specific markers of wound repair. The number of positively stained cells, normalized to area, was quantified in distant lung tissue (n=40 regions of interest from 3 mice). Mean ± SEM (multiple unpaired *t*-tests); * *p*≤0.05, ** *p*<0.01, *** *p*<0.001. Shown are representative images; scale bar = 10µm, inset zoom 2x.
