# Supplementary material for: Metastasis-Associated Wound Repair Promotes Reciprocal Lung Epithelium Activation and Breast Cancer Metastatic Outgrowth
Source: Cancer Res Commun. 2026 Apr 6;6(4):750–68. doi: 10.1158/2767-9764.CRC-25-0459 (PMC13051055; doi:10.1158/2767-9764.CRC-25-0459)
Supplement: Supplementary Figure 3 — Cell activation in metastatic lungs. [file crc-25-0459_supplementary_figure_3_suppsf3.pdf]

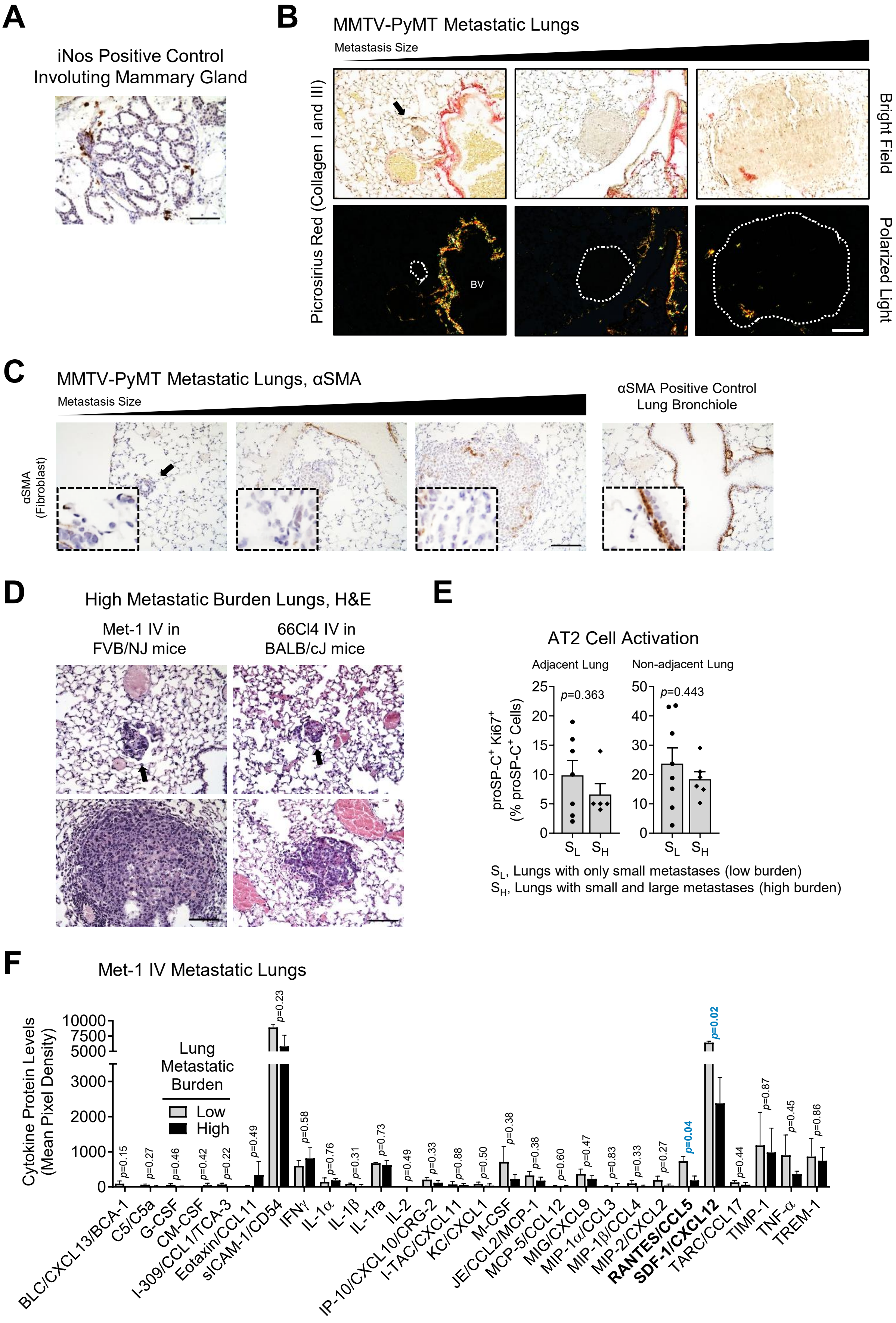

Supplementary Figure 3.

Supplementary Figure 3. Cell activation in metastatic lungs. A, Representative image of iNOS staining in an involuting mouse mammary gland used as a positive control for IHC staining; scale bar = 100µm. **B,** Representative images from MMTV-PyMT metastases of different sizes with black arrow indicating small metastases stained with picrosirius red. Metastases are outlined in white in polarized light images. BV, blood vessel; scale bar = 100µm. **C,** MMTV-PyMT metastatic lungs were stained for the fibroblast activation marker αSMA. Shown are representative images from metastases of different sizes with black arrows indicating small metastases; scale bar = 100µm, inset zoom 4x. **D,** Representative images of H&E stained high metastatic burden lungs from late-stage metastasis models using Met-1 cells in FVB/NJ mice and 66Cl4 cells in BALB/cJ mice; scale bar = 100µm. **E,** Multispectral immunofluorescent staining of MMTV-PyMT metastatic lungs for AT2 cell activation. The percentage of proSP-C-positive cells that are Ki67-positive was quantified in the adjacent and non-adjacent lungs surrounding small metastases from low metastatic burden lungs with only small metastases (S_L) and high metastatic burden lungs with both small and large metastases (S_H) (*n*=14 metastases from 2-3 mice; unpaired *t*-tests), mean $\pm$ SEM. **F,** Cytokine array performed on lungs from mice with a low or high metastatic burden using the late-stage Met-1 metastasis model (*n*=3 mice per group), mean $\pm$ SEM (multiple unpaired *t*-tests with Welch's correction).
