# Supplementary material for: Metastasis-Associated Wound Repair Promotes Reciprocal Lung Epithelium Activation and Breast Cancer Metastatic Outgrowth
Source: Cancer Res Commun. 2026 Apr 6;6(4):750–68. doi: 10.1158/2767-9764.CRC-25-0459 (PMC13051055; doi:10.1158/2767-9764.CRC-25-0459)
Supplement: Supplementary Figure 4 — scRNAseq dissociation gene signature analysis. [file crc-25-0459_supplementary_figure_4_suppsf4.pdf]

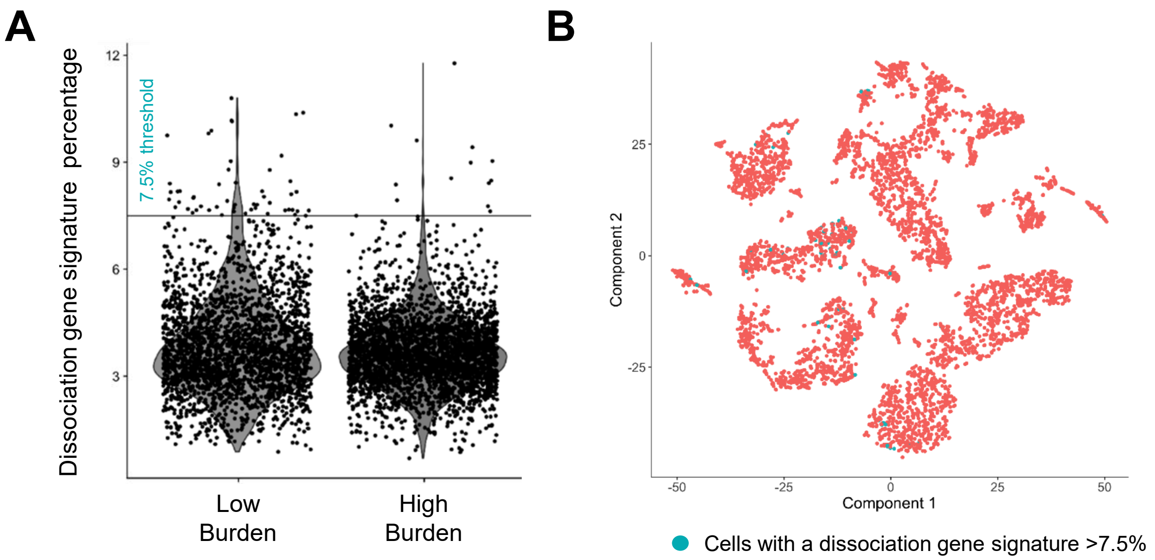

Supplementary Figure 4.

Supplementary Figure 4. scRNAseq dissociation gene signature analysis. Lungs from mice with a low or high metastatic burden using the late-stage Met-1 metastasis model were enzymatically dissociated for downstream scRNAseq analysis (n=1 mouse per group). **A,** The dissociation percentage (PMID: 28960196) was calculated for each cell by examining the counts of each dissociation-related gene (138) relative to the total counts of all genes per cell. 65 cells out of 5504 have a dissociation percentage greater than the published threshold of 7.5%. **B,** t-SNE visualization of cells with a dissociation percentage >7.5% (colored blue). Only 2% of cells from a low metastatic burden lung and 0.4% of cells from high metastatic burden lung have a dissociation signature >7.5%.
