# Supplementary material for: Metastasis-Associated Wound Repair Promotes Reciprocal Lung Epithelium Activation and Breast Cancer Metastatic Outgrowth
Source: Cancer Res Commun. 2026 Apr 6;6(4):750–68. doi: 10.1158/2767-9764.CRC-25-0459 (PMC13051055; doi:10.1158/2767-9764.CRC-25-0459)
Supplement: Supplementary Figure 5 — scRNAseq analysis of metastatic outgrowth in lungs. [file crc-25-0459_supplementary_figure_5_suppsf5.pdf]

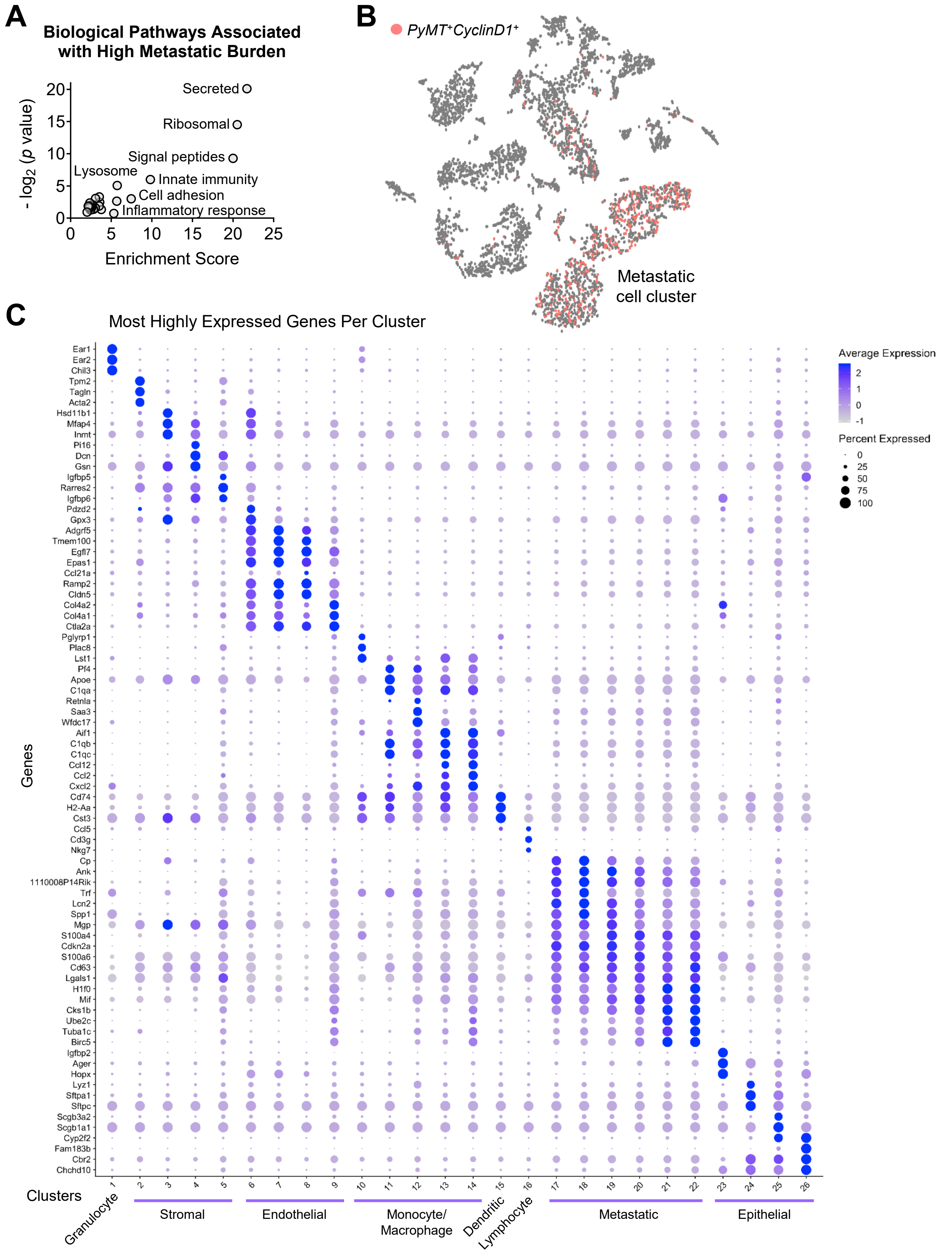

Supplementary Figure 5.

Supplementary Figure 5. scRNAseq analysis of metastatic outgrowth in lungs. Lungs from mice with a low or high metastatic burden using the late-stage Met-1 metastasis model were transcriptionally evaluated using scRNAseq (n=1 mouse per group). **A,** A bulk analysis was performed on the combined data from all cells in lungs with a low metastatic burden compared to cells from lungs with a high metastatic burden. Functional biological pathways associated with metastatic outgrowth were identified using differentially expressed genes. Only pathway clusters with an enrichment score >2 are included. **B,** Identification of the mammary carcinoma metastatic cell population. t-SNE visualization of lung cells clustered by gene expression and colored by co-expression of *PyMT* and *CyclinD1*. **C,** The most highly expressed genes per cell cluster. Average expression defined as average $\log_2$ fold change between one cluster and all other clusters. Percent expression defined as the percentage of cells within each cluster that express each gene.
