# Supplementary material for: Metastasis-Associated Wound Repair Promotes Reciprocal Lung Epithelium Activation and Breast Cancer Metastatic Outgrowth
Source: Cancer Res Commun. 2026 Apr 6;6(4):750–68. doi: 10.1158/2767-9764.CRC-25-0459 (PMC13051055; doi:10.1158/2767-9764.CRC-25-0459)
Supplement: Supplementary Figure 6 — Lung AT2 cells. [file crc-25-0459_supplementary_figure_6_suppsf6.pdf]

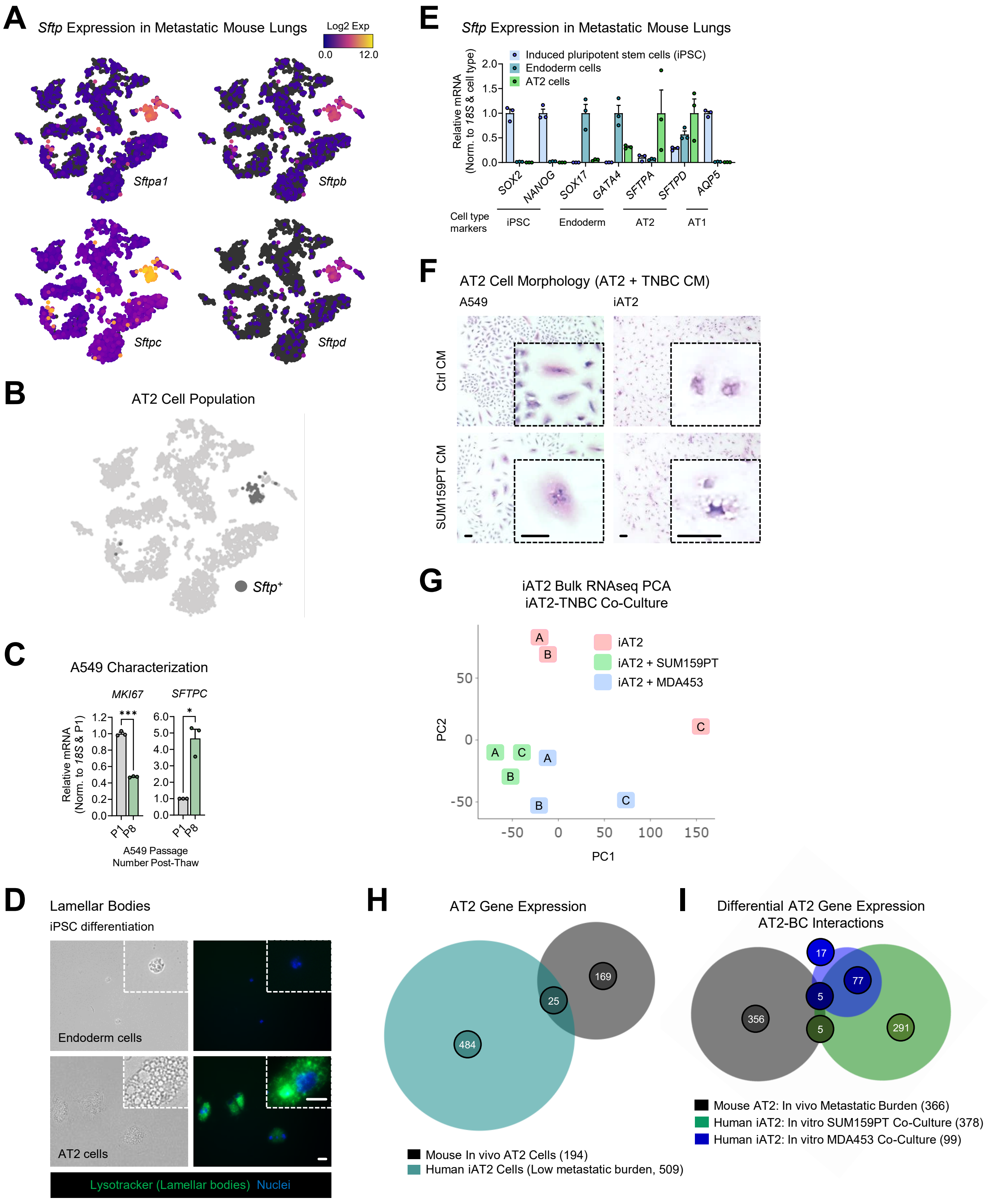

Supplementary Figure 6

Supplementary Figure 6. Lung AT2 cells. A, Lungs from mice with a low or high metastatic burden using the late-stage Met-1 metastasis model were transcriptionally evaluated using scRNAseq (n=1 mouse per group). t-SNE visualization of surfactant protein (*Sftp*) isoform gene expression. **B,** Identification of the AT2 cell population. t-SNE visualization of combined *Sftpa-d* gene expression ($Sftp^{+}$) with a log2 max expression >12. **C,** Gene expression by qPCR of A549 cells following long-term culture (passage 8, P8). Data was normalized to *18S* and early passage A549 cells (passage, P1). Mean ± SEM (unpaired *t*-tests with Welch's correction); * $p \leq 0.05$, *** $p < 0.001$. **D,** iAT2 lamellar bodies were imaged using lysotracker staining following induced pluripotent stem cell (iPSC) differentiation through the endoderm stage to AT2 cells; scale bar = 10µm, inset zoom 3x. **E,** Gene expression by qPCR in iPSC, endoderm, and iAT2 cells for cell type specific markers of differentiation. Data was normalized to *18S* and cell type for each marker; mean ± SEM. **F,** AT2 cells were cultured in 50% conditioned media (CM) from SUM159PT cells or control (Ctrl) media for 3 days. AT2 cell morphology was then examined by H&E. Shown are representative images of cell size and shape; scale bars = 10µm, inset zoom 4x for A549 cells and 6x for iAT2 cells. **G,** Bulk RNAseq was performed on RNA collected from iAT2 cells co-cultured with SUM159PT or MDA453 cells for 5 days. Control iAT2 cells were cultured alone. This principal component analysis (PCA) illustrates the relationship between gene expression data from sample replicates. **H,** Venn diagram comparing genes expressed in AT2 cells in mouse lungs (data from mouse lungs with a low metastatic burden) versus genes expressed in human iAT2 cells cultured alone. **i,** Venn diagram comparing AT2 genes associated with BC cell interactions using differentially expressed genes from mouse AT2 cells in metastatic lungs and human iAT2 cells co-culture with TNBC cells.
