# Supplementary material for: Metastasis-Associated Wound Repair Promotes Reciprocal Lung Epithelium Activation and Breast Cancer Metastatic Outgrowth
Source: Cancer Res Commun. 2026 Apr 6;6(4):750–68. doi: 10.1158/2767-9764.CRC-25-0459 (PMC13051055; doi:10.1158/2767-9764.CRC-25-0459)
Supplement: Supplementary Figure 7 — PDE4 levels and inhibition. [file crc-25-0459_supplementary_figure_7_suppsf7.pdf]

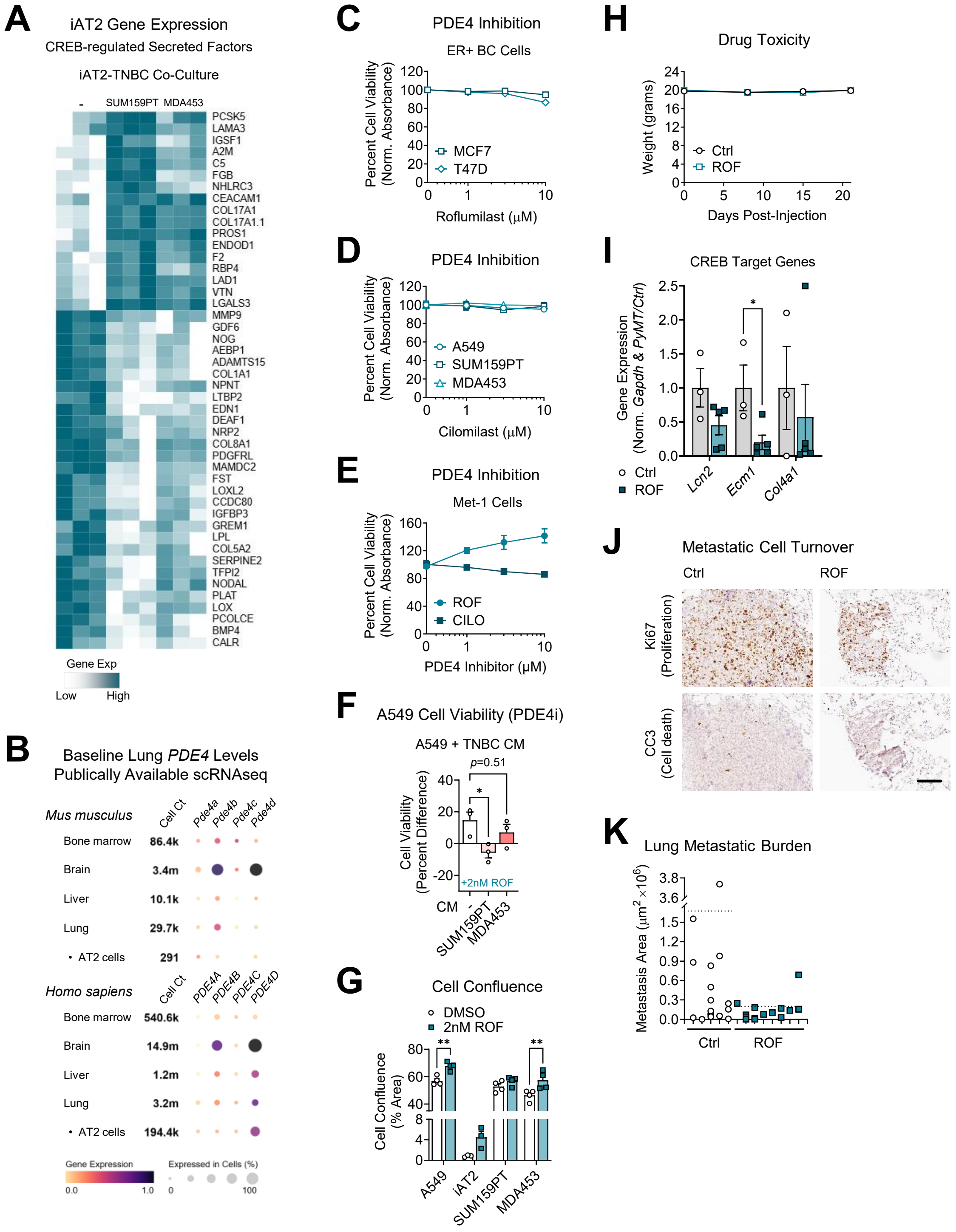

Supplementary Figure 7

Supplementary Figure 7. PDE4 levels and inhibition. A, Heatmap of CREB-regulated secreted factor gene expression in iAT2 cells cultured alone (-) or co-cultured with SUM159PT or MDA453 cells for 5 days. **B,** Publicly available scRNAseq data was queried using the CZ CELLxGENE Discover data platform (PMID: 39607691) for *PDE4* isoform baseline levels in mouse and human tissue and cells. **C,** Percent cell viability was measured by crystal violet assay after 3 day treatment with vehicle DMSO (0) or increasing concentrations of the PDE4 inhibitor roflumilast (ROF). Data was normalized to mean absorbance of DMSO-treated cells; mean ± SEM. **D,** Percent cell viability was measured by crystal violet assay after a 3 day treatment with vehicle DMSO (0) or increasing concentrations of the PDE4 inhibitor cilomilast (CILO). Data was normalized to the mean absorbance of DMSO-treated cells; mean ± SEM. **E,** Percent cell viability was measured in Met-1 mouse carcinoma cells by crystal violet assay after a 3 day treatment with vehicle DMSO (0) or increasing concentrations of ROF or CILO. Data was normalized to the mean absorbance of DMSO-treated cells; mean ± SEM. **F,** A549 cell viability was measured by crystal violet assay following culture with conditioned media (CM) from TNBC cells and treatment with 2nM roflumilast (ROF) for 5 days. The percent difference in cell viability was calculated. Mean ± SEM (one-way ANOVA with Tukey's multiple comparison test); * $p \leq 0.05$. **G,** Cell confluence was examined by crystal violet assay in cells treated with DMSO or 2nM ROF for 5-7 days, depending on the cell line. Mean ± SEM (two-way ANOVA with Sidak's multiple comparison test); ** $p < 0.01$. **H,** Met-1 cells were injected IV into the tail veins of female mice and orally administered 5mg/kg ROF starting 3 days post-injection (n=5-8 mice per group). Mice were treated daily for 3 weeks and drug toxicity was tracked through weekly measurement of mouse weight. Mean ± SEM. **I,** mRNA was isolated from whole lung lysates following ROF treatment in mice and the expression of known CREB-regulated genes was examined by qPCR. Mean ± SEM (unpaired *t*-test); * $p \leq 0.05$. **J,** Metastatic lungs were stained for cell turnover markers Ki67 and cleaved-caspase 3 (CC3) by IHC. Shown are representative images of lung metastases; scale bar = 100µm. **K,** Metastatic lung tissue was collected from ROF treated mice and stained for the Met-1 mammary-specific marker PyMT. Representative data is shown from a single section indicating the number and size of metastases per mouse; each notch on the x-axis represents an individual mouse. The dotted lines represent the means from the full analysis of averages per mouse.
