# Supplementary material for: Metastasis-Associated Wound Repair Promotes Reciprocal Lung Epithelium Activation and Breast Cancer Metastatic Outgrowth
Source: Cancer Res Commun. 2026 Apr 6;6(4):750–68. doi: 10.1158/2767-9764.CRC-25-0459 (PMC13051055; doi:10.1158/2767-9764.CRC-25-0459)
Supplement: Supplementary Figure 8 — PDE4 isoform levels in human lungs. [file crc-25-0459_supplementary_figure_8_suppsf8.pdf]

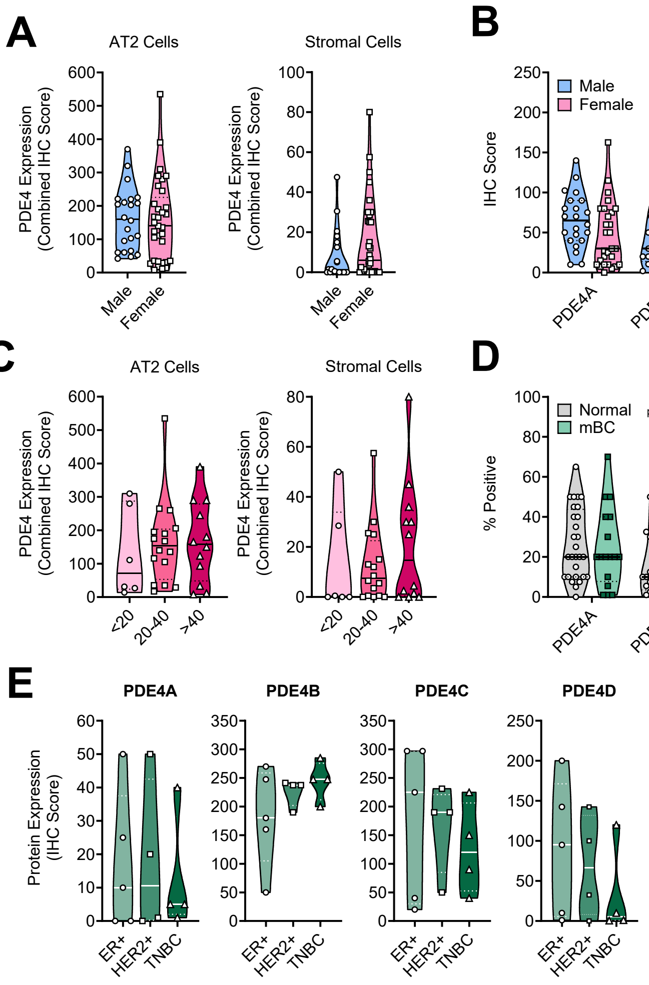

Supplementary Figure 8

Supplementary Figure 8. PDE4 isoform levels in human lungs. Human lung specimens were IHC stained for PDE4 isoforms A-D (see Supplementary Table 1C for specimen details). Protein expression was scored by a clinical pathologist for percentage positive cells and staining intensity. The IHC score was calculated by multiplying the percentage positive and intensity scores, and the combined IHC score was the sum of IHC scores from all four PDE4 isoforms. **A-B,** Lungs from males (*n*=22) and females (*n*=34) were compared. **C,** Lungs from females of increasing age were compared; <20 years of age (*n*=6), 20-40 years of age (*n*=16), and >40 years of age (*n*=12). **D,** The percentage of positively stained cells was scored in lung AT2 cells and the lung stroma from normal (*n*=28) and metastatic (*n*=17) lungs. **E,** Intratumoral PDE4 isoform levels were scored for each metastatic sample and the staining based on BC subtype was compared. For all comparisons the median and quartiles are indicated for each graph (nonparametric Kolmogorov-Smirnov tests for grouped data and nonparametric Kruskal-Wallis test for the intratumoral staining comparison).
